# The International Climate Psychology Collaboration: Climate change-related data collected from 63 countries

**DOI:** 10.1038/s41597-024-03865-1

**Published:** 2024-10-01

**Authors:** Kimberly C. Doell, Boryana Todorova, Madalina Vlasceanu, Joseph B. Bak Coleman, Ekaterina Pronizius, Philipp Schumann, Flavio Azevedo, Yash Patel, Michael M. Berkebile-Wineberg, Cameron Brick, Florian Lange, Samantha J. Grayson, Yifei Pei, Alek Chakroff, Karlijn L. van den Broek, Claus Lamm, Denisa Vlasceanu, Sara M. Constantino, Steve Rathje, Danielle Goldwert, Ke Fang, Salvatore Maria Aglioti, Mark Alfano, Andy J. Alvarado-Yepez, Angélica Andersen, Frederik Anseel, Matthew A. J. Apps, Chillar Asadli, Fonda Jane Awuor, Piero Basaglia, Jocelyn J. Bélanger, Sebastian Berger, Paul Bertin, Michał Białek, Olga Bialobrzeska, Michelle Blaya-Burgo, Daniëlle N. M. Bleize, Simen Bø, Lea Boecker, Paulo S. Boggio, Sylvie Borau, Sylvie Borau, Björn Bos, Ayoub Bouguettaya, Markus Brauer, Tymofii Brik, Roman Briker, Tobias Brosch, Ondrej Buchel, Daniel Buonauro, Radhika Butalia, Héctor Carvacho, Sarah A. E. Chamberlain, Hang-Yee Chan, Dawn Chow, Dongil Chung, Luca Cian, Noa Cohen-Eick, Luis Sebastian Contreras-Huerta, Davide Contu, Vladimir Cristea, Jo Cutler, Silvana D’Ottone, Jonas De keersmaecker, Sarah Delcourt, Sylvain Delouvée, Kathi Diel, Benjamin D. Douglas, Moritz A. Drupp, Shreya Dubey, Jānis Ekmanis, Christian T. Elbaek, Mahmoud Elsherif, Iris M. Engelhard, Yannik A. Escher, Tom W. Etienne, Laura Farage, Ana Rita Farias, Stefan Feuerriegel, Andrej Findor, Lucia Freira, Malte Friese, Neil Philip Gains, Albina Gallyamova, Sandra J. Geiger, Oliver Genschow, Biljana Gjoneska, Theofilos Gkinopoulos, Beth Goldberg, Amit Goldenberg, Sarah Gradidge, Simone Grassini, Kurt Gray, Sonja Grelle, Siobhán M. Griffin, Lusine Grigoryan, Ani Grigoryan, Dmitry Grigoryev, June Gruber, Johnrev Guilaran, Britt Hadar, Ulf J. J. Hahnel, Eran Halperin, Annelie J. Harvey, Christian A. P. Haugestad, Aleksandra M. Herman, Hal E. Hershfield, Toshiyuki Himichi, Donald W. Hine, Wilhelm Hofmann, Lauren Howe, Enma T. Huaman-Chulluncuy, Guanxiong Huang, Tatsunori Ishii, Ayahito Ito, Fanli Jia, John T. Jost, Veljko Jovanović, Dominika Jurgiel, Ondřej Kácha, Reeta Kankaanpää, Jaroslaw Kantorowicz, Elena Kantorowicz-Reznichenko, Keren Kaplan Mintz, Ilker Kaya, Ozgur Kaya, Narine Khachatryan, Anna Klas, Colin Klein, Christian A. Klöckner, Lina Koppel, Alexandra I. Kosachenko, Emily J. Kothe, Ruth Krebs, Amy R. Krosch, Andre P. M. Krouwel, Yara Kyrychenko, Maria Lagomarsino, Julia Lee Cunningham, Jeffrey Lees, Tak Yan Leung, Neil Levy, Patricia L. Lockwood, Chiara Longoni, Alberto López Ortega, David D. Loschelder, Jackson G. Lu, Yu Luo, Joseph Luomba, Annika E. Lutz, Johann M. Majer, Ezra Markowitz, Abigail A. Marsh, Karen Louise Mascarenhas, Bwambale Mbilingi, Winfred Mbungu, Cillian McHugh, Marijn H. C. Meijers, Hugo Mercier, Fenant Laurent Mhagama, Katerina Michalaki, Nace Mikus, Sarah G. Milliron, Panagiotis Mitkidis, Fredy S. Monge-Rodríguez, Youri L. Mora, Michael J. Morais, David Moreau, Kosuke Motoki, Manuel Moyano, Mathilde Mus, Joaquin Navajas, Tam Luong Nguyen, Dung Minh Nguyen, Trieu Nguyen, Laura Niemi, Sari R. R. Nijssen, Gustav Nilsonne, Jonas P. Nitschke, Laila Nockur, Ritah Okura, Sezin Öner, Asil Ali Özdoğru, Helena Palumbo, Costas Panagopoulos, Maria Serena Panasiti, Philip Pärnamets, Mariola Paruzel-Czachura, Yuri G. Pavlov, César Payán-Gómez, Adam R. Pearson, Leonor Pereira da Costa, Hannes M. Petrowsky, Stefan Pfattheicher, Nhat Tan Pham, Vladimir Ponizovskiy, Clara Pretus, Gabriel G. Rêgo, Ritsaart Reimann, Shawn A. Rhoads, Julian Riano-Moreno, Isabell Richter, Jan Philipp Röer, Jahred Rosa-Sullivan, Robert M. Ross, Anandita Sabherwal, Toshiki Saito, Oriane Sarrasin, Nicolas Say, Katharina Schmid, Michael T. Schmitt, Philipp Schoenegger, Christin Scholz, Mariah G. Schug, Stefan Schulreich, Ganga Shreedhar, Eric Shuman, Smadar Sivan, Hallgeir Sjåstad, Meikel Soliman, Katia Soud, Tobia Spampatti, Gregg Sparkman, Ognen Spasovski, Samantha K. Stanley, Jessica A. Stern, Noel Strahm, Yasushi Suko, Sunhae Sul, Stylianos Syropoulos, Neil C. Taylor, Elisa Tedaldi, Gustav Tinghög, Luu Duc Toan Huynh, Giovanni Antonio Travaglino, Manos Tsakiris, İlayda Tüter, Michael Tyrala, Özden Melis Uluğ, Arkadiusz Urbanek, Danila Valko, Sander van der Linden, Kevin van Schie, Aart van Stekelenburg, Edmunds Vanags, Daniel Västfjäll, Stepan Vesely, Jáchym Vintr, Marek Vranka, Patrick Otuo Wanguche, Robb Willer, Adrian Dominik Wojcik, Rachel Xu, Anjali Yadav, Magdalena Zawisza, Xian Zhao, Jiaying Zhao, Dawid Żuk, Jay J. Van Bavel

**Affiliations:** 1 Department of Cognition, Emotion, and Methods in Psychology, Faculty of Psychology, University of Vienna, Vienna, 1010 Austria; 2 Centre for the Advanced Study of Collective Behaviour, University of Konstanz, Konstanz, 78464 Germany; 3 Department of Collective Behaviour, Max-Planck Institute of Animal Behavior, Konstanz, 78464 Germany; 4 Department of Psychology, New York University, New York, 10003 USA; 5 Department of Environmental Social Sciences, Stanford University, Stanford, 94305 USA; 6 Craig Newmark Center for Journalism Ethics and Security, Columbia University, New York, 10018 USA; 7 Institute for Rebooting Social Media, Harvard University, Cambridge, 02138 USA; 8 Department of Psychology, Carl von Ossietzky University of Oldenburg, Oldenburg, 26129 Germany; 9 Department of Psychology, University of Groningen, Groningen, 9712TS The Netherlands; 10 Department of Psychology, University of Amsterdam, Amsterdam, 1018 WT The Netherlands; 11 Department of Psychology, Inland Norway University of Applied Sciences, Elverum, 2418 Norway; 12 Behavioral Economics and Engineering Group, KU Leuven, Leuven, 3000 Belgium; 13 Department of Psychology, Stanford University, Stanford, 94305 USA; 14 San Luis Obispo, 93405 USA; 15 Copernicus Institute of Sustainable Development, Utrecht University, Utrecht, 3584 CB the Netherlands; 16 Research Centre for Environmental Economics, Heidelberg University, Heidelberg, 69115 Germany; 17 Department of Psychology, Universoty of Pittsburgh, Pittsburgh, 15260 USA; 18 School of Public Policy and Urban Affairs, Northeastern University, Boston, 02115 USA; 19 Department of Psychology, Northeastern University, Boston, 02115 USA; 20 Department of Psychology & Neuroscience and Society, Sapienza University of Rome &Italian Institute of Technology, Rome, 179 Italy; 21 Santa Lucia Foundation, IRCCS, Rome, 185 Italy; 22 Department of Philosophy, Macquarie University, Sydney, 2000 Australia; 23 Universidad Peruana Cayetano Heredia; San Martín de Porres, Lima, 15102 Peru; 24 Post-Graduation Program in Linguistics, Federal University of Paraná, Curitiba, 80060150 Brasil; 25 UNSW Business School, University of New South Wales, Sydney, 2052 Australia; 26 Centre for Human Brain Health, School of Psychology, University of Birmingham, Birmingham, B15 2TT UK; 27 Psychology Scientific Research Institute, Baku, Azerbaijan; 28 Kenya Marine and Fisheries Research Institute, Kisumu, 1881- 40100 Kenya; 29 Department of Economics, University of Hamburg, Hamburg, 20146 Germany; 30 Department of Psychology, New York University Abu Dhabi, Abu Dhabi, 129188 United Arab Emirates; 31 Department of Sociology, University of Bern, Bern, 3012 Switzerland; 32 LAPCOS, Université Côte d’Azur, Nice, 6357 France; 33 Center for Social and Cultural Psychology, Université libre de Bruxelles, Brussels, 1050 Belgium; 34 Institute of Psychology, Faculty of Historical and Pedagogical Sciences, University of Wroclaw, Wroclaw, 50-120 Poland; 35 Institute of Psychology, SWPS University, Warsaw, 03-815 Poland; 36 Department of Psychology, Division of Behavioral & Organizational Sciences, Claremont Graduate University, Claremont, 91711 USA; 37 Behavioural Science Institute, Radboud University, Nijmegen, 6500 HE The Netherlands; 38 Department of Strategy and Management, Norwegian School of Economics, Bergen, 5045 Norway; 39 Department of Economic Psychology, Social Psychology and Experimental Methods, Leuphana University Lüneburg, Lüneburg, 21335 Germany; 40 Social and Cognitive Neuroscience Laboratory, Mackenzie Presbyterian University, Sao Paulo, 1241001 Brazil; 41 Institute for Advanced Study in Toulouse, Toulouse, 31000 France; 42 Toulouse Business School, Toulouse, 31000 France; 43 Department of Economics, University of Hamburg, Hamburg, 20146 Hamburg Germany; 44 School of Psychology, University of Birmingham, Birmingham, B15 2TT UK; 45 Department of Psychology, University of Wisconsin–Madison, Madison, 53706 USA; 46 Policy Research Department, Kyiv School of Economics, Kyiv, 2000 Ukraine; 47 Department of Organisation, Strategy, and Entrepreneurship, School of Business and Economics, Maastricht University, Maastricht, 6211 LK The Netherlands; 48 Department of Psychology and Swiss Center for Affective Sciences, University of Geneva, Geneva, 1205 Switzerland; 49 Institute for Sociology of the Slovak Academy of Sciences, Slovak Academy of Sciences, Bratislava, 81364 Slovakia; 50 Psychological Science, Pomona College, Claremont, 91711 USA; 51 Department of Movement Sciences, KU Leuven, Leuven, 3001 Belgium; 52 Escuela de Psicología, Pontificia Universidad Católica de Chile, Santiago, Chile; 53 School of Psychology, Speech, and Hearing, University of Canterbury, University of Canterbury, Christchurch, 8051 New Zealand; 54 Department of Marketing, King’s Business School, King’s College London, London, WC2B 4BG United Kingdom; 55 Department of Management and Marketing, University of Melbourne, Melbourne, 3010 Australia; 56 Department of Biomedical Engineering, Ulsan National Institute of Science and Technology, Ulsan, 44919 Republic of Korea; 57 Department of Marketing, University of Virginia, Darden School of Business, Charlottesville, 22903 USA; 58 Department of Psychology, The Hebrew University, Jerusalem, 9190501 Israel; 59 Department of Psychology, University of Groningen, Groningen, 9712 CP The Netherlands; 60 Center for Social and Cognitive Neuroscience (CSCN), School of Psychology, Universidad Adolfo Ibáñez, Viña del Mar, Chile; 61 School of Management, Canadian University Dubai, Dubai, 117781 UAE; 62 Kieskompas - Election Compass, Amsterdam, 1052XH The Netherlands; 63 Escuela de Psicología, Pontificia Universidad Católica de Chile, Santiago, 8331150 Chile; 64 Department of Developmental, Personality and Social Psychology, Ghent University, Gent, 9000 Belgium; 65 Department of People Management and Organization, Esade Business School, Universitat Ramon Llull, Barcelona, 8034 Spain; 66 LP3C, Université Rennes 2, Rennes, 35000 France; 67 Department of Psychology, Saarland University, Saarbrücken, 66123 Germany; 68 Center for Earth System Research and Sustainability (CEN), University of Hamburg, Hamburg, 20146 Germany; 69 Amsterdam School of Communication Research, University of Amsterdam, Amsterdam, 1018WV The Netherlands; 70 Department of Psychology, University of Latvia, Riga, Latvia; 71 Department of Management, Aarhus University, Aarhus, 8210 Denmark; 72 Department of Psychology, University of Birmingham, Birmingham, B15 2TT United Kingdom; 73 Department of Vision Science, University of Leicester, Leicester, LE1 7RH United Kingdom; 74 Department of Clinical Psychology, Utrecht University, Utrecht, 3508 TC the Netherlands; 75 Institute of Management & Organization, Leuphana University Lüneburg, Lüneburg, 21335 Germany; 76 Department of Political Science & Annenberg School for Communication, University of Pennsylvania, Philadelphia, 19104 USA; 77 Department of Psychology, University of Salzburg, Salzburg, 5020 Salzburg Austria; 78 HEI-Lab: Digital Human-Environment Interaction Labs, Lusófona University, Lisbon, 1700 Portugal; 79 LMU Munich School of Management, LMU Munich, Munich, 80539 Germany; 80 Institute of European Studies and International Relations, Faculty of Social and Economic Sciences, Comenius University Bratislava, Bratislava, 82105 Slovakia; 81 Laboratorio de Neurociencia, Escuela de Negocios, Universidad Torcuato Di Tella, Buenos Aires, C1428 Argentina; 82 School of Global Studies, Thammasat University, Bangkok, 12121 Thailand; 83 Center for Sociocultural Research, HSE University, Moscow, 101000 Russia; 84 Environmental Psychology, Department of Cognition, Emotion, and Methods in Psychology, Faculty of Psychology, University of Vienna, Vienna, A-1010 Austria; 85 Institute for Management and Organization, Leuphana University Lüneburg, Lüneburg, 21335 Germany; 86 Macedonian Academy of Sciences and Arts, Skopje, 1000 North Macedonia; 87 Faculty of Philosophy, Institute of Psychology, Faculty of Philosophy, Jagiellonian University, Krakow, 30-060 Poland; 88 Jigsaw, Google, New York, 10011 USA; 89 Harvard Business School, Harvard University, Boston, 2163 USA; 90 Department of Psychology, Harvard University, Cambridge, 2138 USA; 91 Digital Data and Design institute at Harvard, Harvard University, Allston, 2134 USA; 92 School of Psychology and Sport Science, Anglia Ruskin University, Cambridge, CB1 1PT UK; 93 Psychosocial Science, University of Bergen, Bergen, 5007 Norway; 94 Cognitive and Behavioral Neuroscience Laboratory, University of Stavanger, Stavanger, 4021 Norway; 95 Department of Psychology and Neuroscience, University of North Carolina, Chapel Hill, Chapel Hill 27599 USA; 96 Department of Psychology, Ruhr University Bochum, Bochum, 44801 Germany; 97 Department of Psychology, University of Limerick, Limerick, V94T9PX Ireland; 98 Department of Psychology, University of York, York, YO10 5DD UK; 99 Department of Personality Psychology, Yerevan State University, Yerevan, “0025” Armenia; 100 Department of Psychology and Neuroscience, University of Colorado Boulder, Boulder, 80309 USA; 101 Division of Social Sciences, University of the Philippines Visayas, Miagao, 5023 Philippines; 102 Baruch Ivcher School of Psychology, Reichman University, Herzliya, 4610101 Israel; 103 Faculty of Psychology, University of Basel, Basel, 4055 Switzerland; 104 Department of Psychology, University of Oslo, Oslo, 373 Norway; 105 Nencki Institute of Experimental Biology, Polish Academy of Sciences, Warsaw, 02-093 Poland; 106 School of Psychology, University of Sussex, Falmer, BN1 9RH UK; 107 Anderson School of Management, University of California, Los Angeles, Los Angeles 90095 USA; 108 School of Economics & Management, Kochi University of Technology, Kami City, 782-8502 Japan; 109 School of Psychology, Speech and Hearing, University of Canterbury, Christchurch, 8051 New Zealand; 110 Department of Business Administration, University of Zurich, Zurich, 8032 Switzerland; 111 Department of Psychology, Universidad Nacional de San Antonio Abad del Cusco, Cusco, 800 Peru; 112 Department of Media and Communication, City University of Hong Kong, Hong Kong, 999077 China; 113 Department of Psychology, Japan Women’s University, Tokyo, 1128681 Japan; 114 Graduate School of Education, Tohoku University, Sendai, 9808576 Japan; 115 Department of Psychology, Seton Hall University, South Orange, 7079 USA; 116 Department of Psychology, Faculty of Philosophy, University of Novi Sad, Novi Sad, 21000 Serbia; 117 Doctoral School of Social Sciences, Nicolaus Copernicus University, Toruń, 87-100 Poland; 118 Green Dock, Hostivice, 25301 Czech Republic; 119 Faculty of Social Sciences, Tampere University, Tampere, 33100 Finland; 120 INVEST Research Flagship, University of Turku, Turku, 20014 Finland; 121 Institute of Security and Global Affairs, Leiden University, The Hague, 2511DP The Netherlands; 122 Erasmus School of Law, Erasmus University Rotterdam, Rotterdam, 3062PA The Netherlands; 123 Shamir Research Institute, University of Haifa, Haifa, 3498838 Israel; 124 Department of Learning and Instructional Sciences, University of Haifa, Haifa, 3498838 Israel; 125 Deparment of Economics, American University of Sharjah, Sharjah, 26666 UAE; 126 School of Psychology, Deakin University, Geelong, 3216 Australia; 127 School of Philosophy, Australian National University, Canberra, 2600 Australia; 128 Department of Psychology, Norwegian University of Science and Technology, Trondheim, 7049 Norway; 129 Department of Management and Engineering, Linköping University, Linköping, 58183 Sweden; 130 Academic and Research Laboratory of Neurotechnology, Ural Federal University, Ekaterinburg, 620075 Russia; 131 Department of Experimental Psychology, Ghent University, Ghent, 9000 Belgium; 132 Department of Psychology, Cornell University, Ithaca, 14850 USA; 133 Departments of Political Science and Communication Science, Vrije Universiteit Amsterdam, Amsterdam, 1081HV The Netherlands; 134 Department of Psychology, University of Cambridge, Cambridge, CB2 3EL UK; 135 Psychology of Sustainability and Behavior Change, University of Basel, Basel, 4055 Switzerland; 136 Management & Organizations, Stephen M. Ross School of Business, University of Michigan, Ann Arbor, 48105 USA; 137 John E. Walker Department of Economics, Clemson University, Clemson, 29634 USA; 138 Andlinger Center for Energy and the Environment, Princeton University, Princeton, 8544 USA; 139 School of Business and Creative Industries, University of the Sunshine Coast, Queensland, 4556 Australia; 140 Department of Philosophy, Macquarie University, Sydney, 2109 Australia; 141 Marketing, Bocconi University, Milan, 20136 Italy; 142 Department of Communication Science, Vrije Universiteit Amsterdam, Amsterdam, 1081HV The Netherlands; 143 Institute of Management and Organization, Leuphana University of Lüneburg, Lueneburg, 21337 Germany; 144 MIT Sloan School of Management, Massachusetts Institute of Technology, Cambridge, MA 2139 USA; 145 Department of Psychology, University of British Columbia, Vancouver, V6T 1Z4 Canada; 146 Tanzanian Fisheries Research Institute, Mwanza, Tanzania; 147 Department of Psychology, Simon Fraser University, Burnaby, BC V5A 1S6 Canada; 148 Department of Social, Organizational, & Economic Psychology, University of Hildesheim, Hildesheim, 31141 Germany; 149 Department of Environmental Conservation, University of Massachusetts Amherst, Amherst, 1003 USA; 150 Department of Psychology, Georgetown University, Washington, 20057 USA; 151 Research Centre for Greenhouse Gas Innovation (RCGI), University of São Paulo, São Paulo, 05508-030 Brazil; 152 Department of Social Psychology, Institute of Psychology, University of São Paulo, São Paulo, 05508-030 Brazil; 153 National Fisheries Resources Research Institute, Jinja, Uganda; 154 Department of Civil and Water Resources Engineering School of Engineering and Technology, Sokoine University of Agriculture, Morogoro, Tanzania; 155 Department of Psychology, University of Limerick, Limerick, V94 T9PX Ireland; 156 Department of Communication Science, University of Amsterdam, Amsterdam, 1001 NG the Netherlands; 157 Institut Jean Nicod, Département d’études cognitives, ENS, EHESS, PSL University, CNRS, Paris, 75005 France; 158 Tanzania Fisheries Research Institute, Mwanza, Tanzania; 159 Royal Holloway, University of London, Egham, TW200EX United Kingdom; 160 School of Culture and Society - Interacting Minds Centre, Aarhus University, Aarhus, 8000 Denmark; 161 Department of Psychology, Universidad Peruana Cayetano Heredia, Lima, 2002 Peru; 162 Fonds de la Recherche Scientifique, Brussels, 1050 Belgium; 163 Center for Social and Cultural Psychology, Université libre de Bruxelles, Brussels, 1312 Belgium; 164 Amazon, Seattle, 98109 USA; 165 School of Psychology, University of Auckland, Auckland, 1010 New Zealand; 166 Department of Management, The University of Tokyo, Tokyo, 113-8654 Japan; 167 Department of Psychology, University of Cordoba, Cordoba, 14071 Spain; 168 Département d’études cognitives, Institut Jean Nicod ENS, EHESS, PSL University, CNRS, Tokyo, 113-8654 Japan; 169 Comisión Nacional de Investigaciones Científicas y Técnicas (CONICET), Buenos Aires, Argentina; 170 Laboratorio de Neurociencia, Escuela de Negocios, Universidad Torcuato Di Tella, Buenos Aires, C1428 CABA Argentina; 171 University of Economics HCMC (UEH), Ho Chi Minh City, Vietnam; 172 College of Management, National Kaohsiung University of Science and Technology, Kaohsiung, 800 Taiwan; 173 Department of Psychology and Dyson School of Applied Economics and Management, Cornell University, Ithaca, 14850 USA; 174 Department of Clinical Neuroscience, Karolinska Institutet, Stockholm, 17177 Sweden; 175 Department of Psychology, Stockholm University, Stockholm, 11419 Sweden; 176 Swedish National Data Service, Gothenburg University, Gothenburg, 41390 Sweden; 177 Department of Psychology and Behavioural Sciences, Aarhus University, Aarhus, 8000 Denmark; 178 Department of Psychology, Kadir Has University, İstanbul, 34083 Turkey; 179 Department of Psychology, Marmara University, İstanbul, 34722 Turkey; 180 Department of Psychology, Üsküdar University, İstanbul, 34662 Turkey; 181 Department of Economics and Business, Universitat Pompeu Fabra, Barcelona, 8005 Spain; 182 Department of Political Science, Northeastern University, Boston, 2115 USA; 183 IRCCS, Santa Lucia Foundation, Rome, 142 Italy; 184 Department of Psychology, Sapienza University of Rome, Rome, 185 Italy; 185 Department of Clinical Neuroscience, Division of Psychology, Karolinska Institutet, Stockholm, 171 77 Sweden; 186 Penn Center for Neuroaesthetics, University of Pennsylvania, Philadelphia, 19104 USA; 187 Institute of Psychology, University of Silesia in Katowice, Katowice, 40-007 Poland; 188 Institute of Medical Psychology and Behavioral Neurobiology, University of Tuebingen, Tuebingen, 72076 Germany; 189 Dirección Académica, Universidad Nacional de Colombia, Sede de La Paz, Cesar, Colombia; 190 HEI-Lab: Digital Human-Environment Interaction Labs, Lusófona University, Lisbon, Portugal; 191 Institute of Management and Organization, Leuphana University Lueneburg, Lueneburg, 21337 Germany; 192 School of Business, International University, Vietnam National University Ho Chi Minh City, Ho Chi Minh City, 700000 Vietnam; 193 Department of Psychology, Durham University, Durham, DH1 3LE UK; 194 Department of Psychobioloogy and Methodology of Heath Sciences, Universitat Autònima de Barcelona, Barcelona, 8193 Spain; 195 Center for Health and Biological Sciences, Mackenzie Presbyterian University, São Paulo, 01221-040 Brazil; 196 Department of Psychology, Georgetown University, Washington, DC 20057 USA; 197 Center for Computational Psychiatry, Icahn School of Medicine at Mount Sinai, New York, 10029 USA; 198 Faculty of Medicine, Universidad Cooperativa de Colombia, Villavicencio, Colombia; 199 Department of Bioethics, El Bosque University, Bogotá, DC Colombia; 200 Department of Psychology, Faculty for Social and Educational Sciences, Norwegian University of Science and Technology, Trondheim, 7491 Norway; 201 Department of Psychology and Psychotherapy, Witten/Herdecke University, Witten, 58455 Germany; 202 Department of Psychology, University of California, Los Angeles, Los Angeles 90095 USA; 203 Department of Psychological and Behavioural Science, London School of Economics and Political Science, London, WC2A 2AE UK; 204 Japan Society for the Promotion of Science, Tokyo, 1020083 Japan; 205 Faculty of Science and Engineering, Waseda university, Tokyo, 1658555 Japan; 206 Institute of Psychology, University of Lausanne, Lausanne, 1015 Switzerland; 207 Department of Management, Prague University of Economics and Business, Prague, 13067 Czech Republic; 208 Department of People Management and Organization, Universitat Ramon Llull, Esade Business School, Barcelona, 8034 Spain; 209 School of Economics & Finance, University of St Andrews, St Andrews, KY16 9AJ UK; 210 School of Philosophical, Anthropological and Film Studies, University of St Andrews, St Andrews, KY16 9AJ UK; 211 Department of Communication, Amsterdam School of Communication Research, University of Amsterdam, Amsterdam, 1018WV The Netherlands; 212 Department of Psychology, Widener University, Chester, 19013 USA; 213 Department of Cognitive Psychology, Universität Hamburg, Hamburg, 20146 Germany; 214 Department of Nutritional Sciences, University of Vienna, Vienna, 1090 Austria; 215 Harvard Business School, Harvard university, Boston, 2163 USA; 216 Department of Social Psychology, Reichman University (RUNI), Herzliya, 4610101 Israel; 217 Research Center for Digital Transformation, Leuphana University Lüneburg, Lüneburg, 21335 Germany; 218 Department of Biomedicine, Aarhus University, Aarhus, 8000 Denmark; 219 Danish Research Institute of Translational Neuroscience (DANDRITE), Aarhus University, Aarhus, 8000 Denmark; 220 Faculty of Psychology and Educational Sciences, University of Geneva, Geneva, 1205 Switzerland; 221 Swiss Center for Affective Sciences, University of Geneva, Geneva, 1205 Switzerland; 222 Department of Psycholog and Neuroscience, Boston College, Chestnut Hill, 2467 USA; 223 Faculty of Philosophy, Ss. Cyril and Methodius University in Skopje, Skopje, 1000 Republic of North Macedonia; 224 Faculty of Philosophy, University of Ss. Cyril and Methodius in Trnava, Trnava, 917 01 Slovakia; 225 School of Medicine and Psychology, Australian National University, Canberra, 200 Australia; 226 Department of Psychology, University of Virginia, Charlottesville, 22902 USA; 227 Faculty of Social Sciences/Psychology, Tampere University, Tampere, FI-33014 Finland; 228 Department of Psychology, Pusan National University, Busan, 46241 Republic of Korea; 229 Psychology and Neuroscience; Schiller Institute for Integrated Science and Society, Boston College, Brighton, 2135 USA; 230 UQ Business School, University of Queensland, Brisbane, 4067 Australia; 231 Department of Developmental Psychology and Socialisation, University of Padova, Padua, 35131 Italy; 232 School of Business and Management, Queen Mary University of London; London, E1 4NS London, United Kingdom; 233 Institute for the Study of Power, Crime, and Society | Department of Law & Criminology, Royal Holloway, University of London, Egham, TW200EX United Kingdom; 234 Department of Psychology, Üsküdar University, Istanbul, 34664 Turkey; 235 Department of Public and International Affairs, City University of Hong Kong, Kowloon, 999077 Hong Kong; 236 Division of Public Policy, The Hong Kong University of Science and Technology, Kowloon, 999078 Hong Kong; 237 School of Psychology, University of Sussex, Falmer, BN19QH United Kingdom; 238 Institute of Pedagogy, Faculty of Historical and Pedagogical Sciences, University of Wroclaw, Wroclaw, 50-120 Poland; 239 Research Department, The South Ural University of Technology, Chelyabinsk, 454052 Russian Federation; 240 Laboratory of Interdisciplinary Space Studies, School for Environmental and Social Studies, Tyumen State University, Tyumen, 625003 Russian Federation; 241 Department of Psycholgy, Cambridge University, Cambridge, CB2 3EB UK; 242 Department of Medical and Clinical Psychology, Tilburg University, Tilburg, 5037 AB the Netherlands; 243 Behavioural Science Institute, Radboud University, Nijmegen, 6500 HE the Netherlands; 244 Department of Psychology, University of Latvia, Riga, 1083 Latvia; 245 Division of Psychology, Linköping University, Linköping, 58183 Sweden; 246 Faculty of Social Sciences, Charles University, Prague, 11000 Czech Republic; 247 Kenya Marine and Fisheries Research Institute, Kisumu, 1881-40100 Kenya; 248 Department of Sociology, Stanford University, Stanford, 94305 USA; 249 Faculty of Philosophy and Social Sciences, Nicolaus Copernicus University, Toruń, 87-100 Poland; 250 Department of Humanities and Social Sciences, Climate and Energy Policy Research Lab, Indian Institute of Technology Kanpur, Kanpur, 208016 India; 251 School of Computing, Engineering and Mathematical Sciences, La Trobe University Melbourne, Melbourne, 3086 Australia; 252 Kellogg School of Management, Northwestern University, Evanston, 60208 USA; 253 Institute for Resources, Environment and Sustainability, University of British Columbia, Vancouver, V6T 1Z4 Canada; 254 Faculty of Psychology, University of Warsaw, Warsaw, 00-183 Poland; 255 Department of Psychology, New York University; New York University, New York, 10003 USA; 256 Center for Neural Science, New York University; New York University, New York, 10003 USA; 257 Norwegian School of Economics, Bergen, Norway

**Keywords:** Human behaviour, Climate-change mitigation

## Abstract

Climate change is currently one of humanity’s greatest threats. To help scholars understand the psychology of climate change, we conducted an online quasi-experimental survey on 59,508 participants from 63 countries (collected between July 2022 and July 2023). In a between-subjects design, we tested 11 interventions designed to promote climate change mitigation across four outcomes: climate change belief, support for climate policies, willingness to share information on social media, and performance on an effortful pro-environmental behavioural task. Participants also reported their demographic information (e.g., age, gender) and several other independent variables (e.g., political orientation, perceptions about the scientific consensus). In the no-intervention control group, we also measured important additional variables, such as environmentalist identity and trust in climate science. We report the collaboration procedure, study design, raw and cleaned data, all survey materials, relevant analysis scripts, and data visualisations. This dataset can be used to further the understanding of psychological, demographic, and national-level factors related to individual-level climate action and how these differ across countries.

## Background & Summary

Climate change is a global threat to human thriving^1^. Combating it more effectively requires massive changes at the individual, collective, and system levels^1–5^. Research has investigated many factors, including the antecedents, associations, and underlying processes related to climate change mitigation (e.g., beliefs, behaviours)^6–10^. However, much of this research has been conducted on Western, highly Educated samples from Industrialized, Rich, and Democratic countries (i.e., WEIRD), which limits the generalizability of the findings^11^. Further, research typically uses correlational methods, precluding an understanding of what factors actually cause climate action. Given that climate change presents a global threat, it is critical to better understand these factors, and how they impact climate change mitigation across the globe^12^.

This manuscript describes the data gathered for the **International Collaboration to Understand Climate Action** (https://bit.ly/3VszDE9)^13^. This collaboration included 258 researchers and data collected from 63 countries across the globe between July 2022 and July 2023 (Supplemental Figure S1). A total of 83,927 participants signed up to participate, of which 59,508 eligible participants are presented in this manuscript (see below for the inclusion/exclusion criteria). When designing this project, our primary aim was to develop and test 11 expert crowd-sourced interventions (described in Table 1) designed to promote climate change mitigation, assessed by multiple outcome variables, in as many countries as possible (the preregistration for this main aim can be found at https://aspredicted.org/blind.php?x=W83_WTL). The outcomes included belief in climate change, support for climate mitigation policies, willingness to share climate-relevant information on social media, and a modified version of the Work for Environmental Protection Task (WEPT; explained further below)^14^.

**Table 1:** Intervention names, descriptions, and relevant statistics.

Intervention	Description	Relevant Statistics
Dynamic Social Norms^28^	Informs participants of how norms are changing and “more and more people are becoming concerned about climate change”, suggesting that people should take action.	Median duration (SD): 49.50 (126.28) Raw N: 6820 Cleaned N (%): 5172 (75.84)
Work Together Norm^29^	Combines referencing a social norm (i.e., “a majority of people are taking steps to reduce their carbon footprint”) with an invitation to “join in” and work together with fellow citizens toward this common goal.	Duration: 162.97 (253.76) Raw N: 6835 Cleaned N: 5160 (75.49)
Effective Collective Action^30,31^	Features examples of successful collective action that have had meaningful effects on climate policies (e.g., protests) or have solved past global issues (e.g., the restoration of the ozone layer).	Duration: 154.34 (321.56) Raw N: 6818 Cleaned N: 5169 (75.81)
Psychological Distance^32^	Frames climate change as a proximal risk by using examples of recent natural disasters caused by climate change in each participants’ nation and prompts them to write about the climate impacts on their community.	Duration: 289.55 (337.26) Raw N: 6717 Cleaned N: 4737 (70.52)
System Justification^33^	Frames climate change as threatening to the way of life to each participant’s nation, and makes an appeal to climate action, as the patriotic response.	Duration: 80.17 (152.10) Raw N: 6854 Cleaned N: 5179 (75.56)
Future-Self Continuity^34^	Emphasizes identification with future selves by asking each participant to project themselves into the future and write a letter addressed to themselves in the present, describing the actions they would have wanted to take regarding climate change.	Duration: 258.02 (523.07) 6491 Cleaned N: 4226 (65.11)
Negative Emotions^35,36^	Exposes participants to ecologically valid scientific facts regarding the impacts of climate change framed in a ‘doom and gloom’ style of messaging that were drawn from different real-world news and media sources.	Duration: 213.10 (295.31) Raw N: 6778 Cleaned N: 5167 (76.23)
Pluralistic Ignorance^37^	Presents real public opinion data collected by the United Nations that shows what percentage of people in each participant’s country agree that climate change is a global emergency.	Duration: 36.89 (1055.17) Raw N: 6876 Cleaned N: 5172 (75.22)
Letter to Future Generation^38,39^	Emphasizes how one’s current actions impact future generations by asking participants to write a letter to a socially close child who will read it in 25 years when they are an adult, describing current actions towards ensuring a habitable planet.	Duration: 346.20 (490.72) Raw N: 6404 Cleaned N: 4044 (63.15)
Binding Moral Foundations^40^	Invokes authority (e.g., “From scientists to experts in the military, there is near universal agreement”), purity (e.g., keep our air, water, and land pure), and ingroup-loyalty (e.g., “it is the American solution”) moral foundations.	Duration: 13.48 (58.64) Raw N: 6877 Cleaned N: 5092 (74.04)
Scientific Consensus^22^	Informs participants that “99% of expert climate scientists agree that the Earth is warming, and climate change is happening, mainly because of human activity”.	Duration: 11.76 (272.47) Raw N: 6892 Cleaned N: 5296 (76.84)
Control Condition	Participants read a brief paragraph that was unrelated to climate change (i.e., a short paragraph from the novel “Great Expectations” by Charles Dickens).	Duration: 70.92 (247.49) Raw N: 6847 Cleaned N: 5094 (74.40)

Each primary outcome was chosen due to its theoretical and practical relevance (see^12^). Briefly, belief in climate change is a key antecedent of pro-environmental intentions, behaviour, and policy support^6^. Public support for a given policy is the top predictor of policy adoption, especially within the realm of climate change^3,15^. Discussing and sharing information about climate change with one’s peers is an essential step in addressing climate change^12,16^, thus we also added the willingness to share information on social media variable. Finally, real, effortful pro-environmental behaviour is needed in order to fight climate change, thus we added the WEPT, which is a web-based task that allows us to measure the amount of effort participants are willing to exert to help protect the environment^14^.

In order to easily assess the average impact of the interventions on each of the main outcome variables (beliefs, policy support, social media sharing, and the WEPT), varied across multiple demographics including nationality, political ideology, age, gender, education, income level and perceived level of socioeconomic status, we provide an easy to use and disseminate webtool called the Climate Intervention Webapp: https://climate-interventions.shinyapps.io/climate-interventions/.

**Fig. 1:**
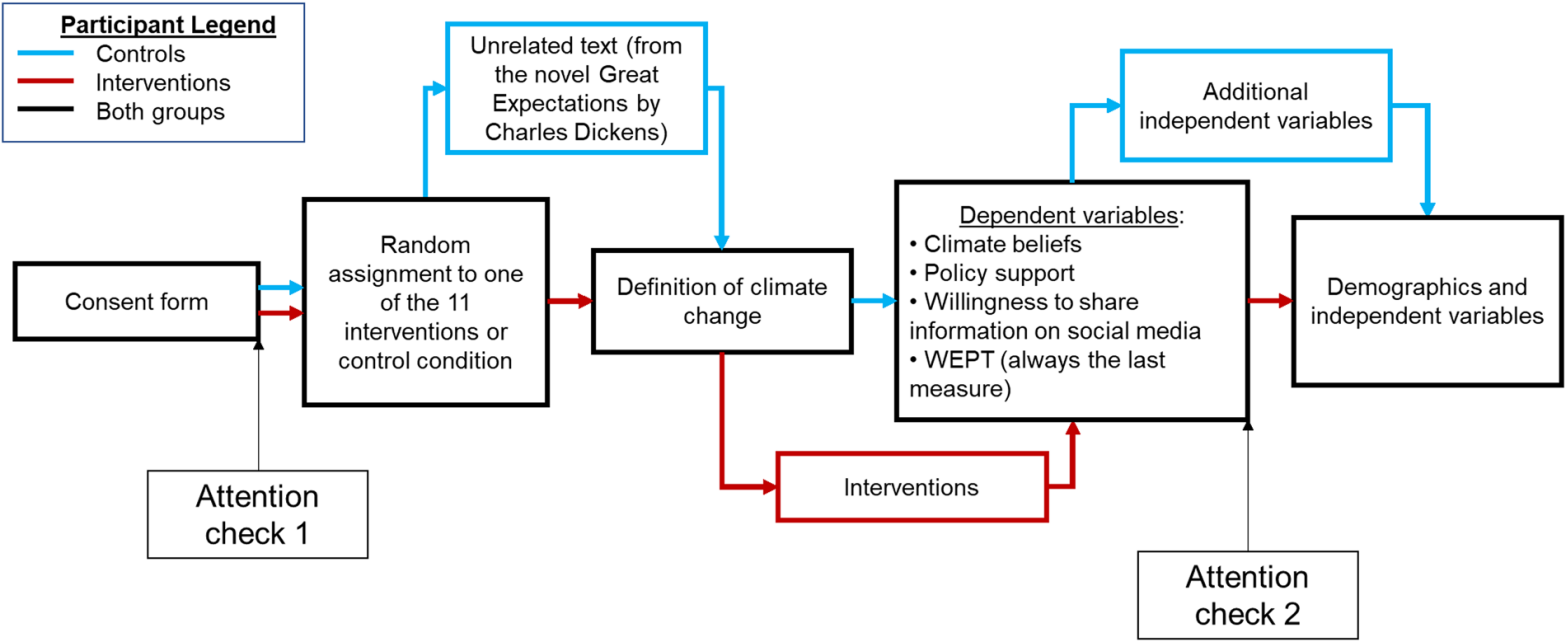
Schematic overview of the survey flow. The pathways for the control participants are shown in blue, and the intervention participants are shown in red.

Our secondary aim was to maximise the utility of the data collected. To do that and also keep the survey length similar across all conditions, participants in the no-intervention control condition responded to numerous additional variables. This included items such as trust in climate scientists, degree of environmentalist identity^17^, and second-order climate beliefs (a full list of included items is reported below). A schematic overview of the survey design is shown in Figure 1.

Due to the richness of this dataset^13^, there are a multitude of secondary analyses that are possible. For example, the effectiveness of the interventions can be explored across socio-political variables^18^, individualism-collectivism^19^, or a number of other factors^20^.

**Fig. 2:**
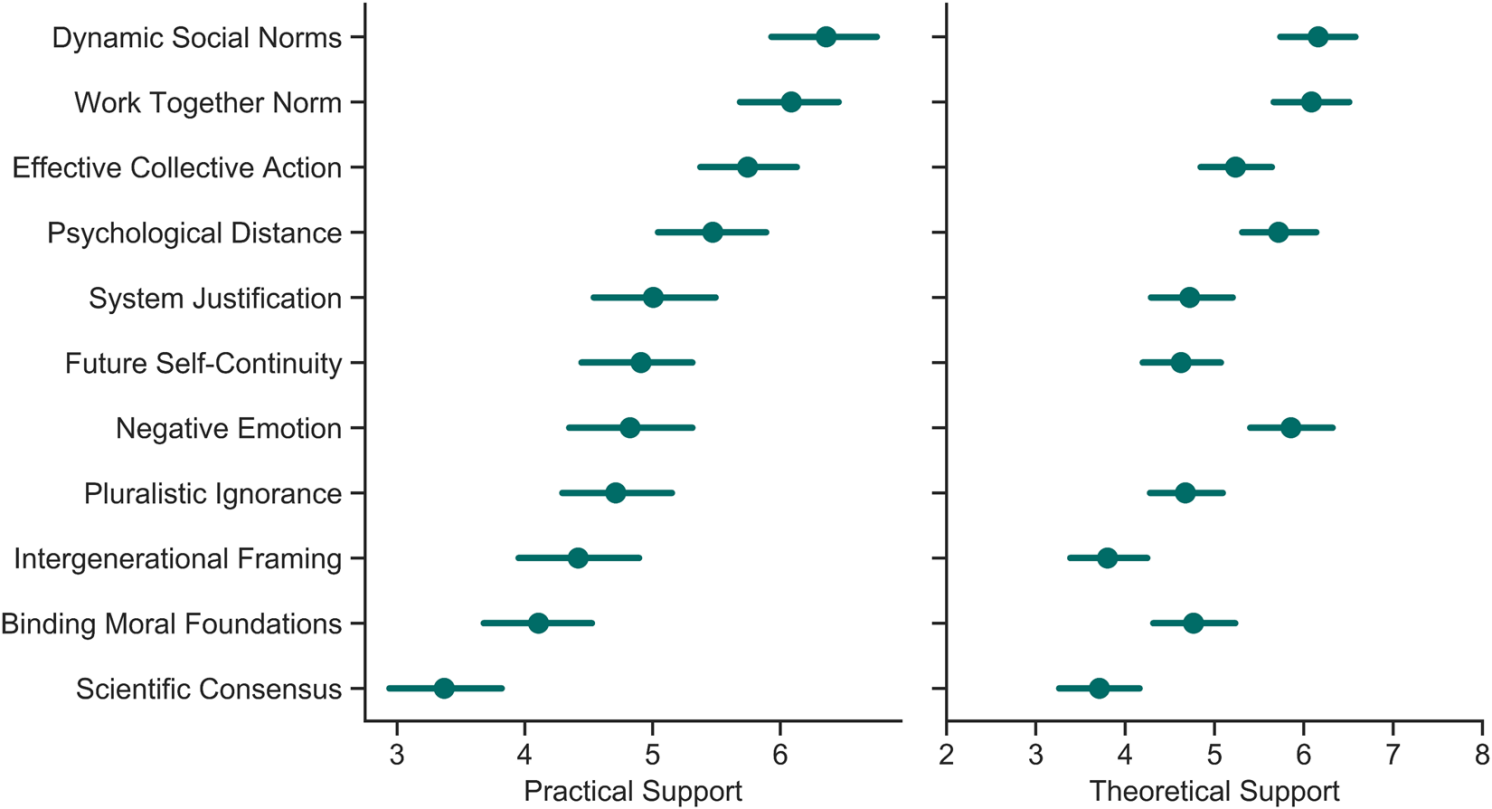
Average support of each crowdsourced intervention. Support was ranked by a sample of 188 behavioural scientists (coauthors on the current paper) who were asked to rate the interventions on perceived efficiency (practical support) and theoretical value (theoretical support). Error bars are bootstrapped confidence intervals around the mean. The mean is a mean rank, where the rank ordinals are defined such that 10 means most support and 0 means least support.

In addition to the above-mentioned participant data, we also present data from an intervention tournament which was conducted before the study, where collaborators submitted interventions they wished to see tested in this international context (more information can be found in the section “Intervention tournament”, below). We received 36 submissions, which were sorted and cleaned by the organisational team (see below for more). The remaining 11 interventions were then rank-ordered by 188 of our collaborators in terms of their practical and theoretical support (Figure 2). Given the high levels of support from our collaborators for all interventions, we decided to include all 11 interventions in the main project.

## Methods

### Collaboration Procedure

In early November of 2021, the organising team (i.e., K. C. Doell, M. Vlasceanu, & J. J. Van Bavel) announced a call for collaboration (https://manylabsclimate.wordpress.com/call-for-collaboration/) on social media, via personal networks, and by posting on various mailing lists and forums.

We announced that researchers could join this collaboration by contributing in one of three ways: (1) collect data (i.e., >500 responses), (2) propose and design an intervention included in the final study, and/or (3) financially contribute to the acquisition of data (i.e., >500 responses) in a country not yet covered in the collaboration. We aimed to limit the cost of collaboration in two specific ways. First, we prioritised creating a relatively short survey (i.e., less than 20 minutes total). This meant the intervention designers had to create interventions that took no more than 5 minutes. Second, while we strongly encouraged data-collection collaborators to recruit representative samples from market research agencies, representative data was not required (see the Participant section for more details).

### Intervention tournament

We invited all collaborators to submit proposals for interventions to be tested via the survey platform Qualtrics (https://www.qualtrics.com/). They were required to submit a short abstract that outlined their intervention and included any relevant references. They were also required to calculate the effect sizes of each intervention based on previous work. Finally, they were asked to consider time constraints (i.e., no more than 5 minutes).

We received 36 proposed interventions, which two authors from the organisational team screened (who were blinded to the intervention authors). The screening procedure involved removing interventions that were not feasible in an international context (e.g., removing proposals including videos that needed to be translated), relevance for the dependent variables, and theoretical support from prior work (quantified by previously reported effect sizes). We also aggregated similar interventions and duplicates. We identified 11 unique and feasible interventions. We then asked all collaborators to read the short summaries of the interventions and rank-order them based on their practical support (i.e., Please rank the following climate interventions in order of their practical support (will it be successful?) from *1* = *“most important”, to 11* = *“least important*”) and theoretical support (Please rank the following climate interventions (their descriptions are above) in order of their theoretical importance from *1* = *“most important”, to 11* = *“least important”*). We obtained 188 responses from our collaborators in January 2022 (Figure 2). The Qualtrics file, and the data from this survey can be found in the “ClimateManylabs_InterventionTournamentVote” folder in the data repository.

### Intervention design

Given high levels of support for all interventions (Figure 2), we tested all 11 interventions in the main study^12^. We then contacted the collaborators whose interventions had been selected to be included to coordinate the intervention implementation and programming on the Qualtrics survey platform. All interventions went through two rounds of reviews. First, the organisational team gave the intervention designers feedback on their submissions and allowed them time to address the comments. After receiving the revised interventions, we contacted expert researchers who had published relevant theoretical work, asking them to review each intervention’s implementation critically. For example, Professor John Jost reviewed the System Justification intervention^21^. Professor Sander van der Linden reviewed the Scientific Consensus intervention^22^. This process was iterated for each of the 11 interventions.

Finally, the organisational team asked all collaborators from around the world for additional feedback on the entire survey, including all interventions, demographics, and independent variables. This was to improve the overall quality and to help reduce any American-centric researcher biases that may have influenced the original survey.

This revision process lasted until the end of May 2022, when we started piloting the final version of the study, on a sample of 723 participants collected in the United States (M_age_ = 43.6; SD_age_ = 15.7; 52% women, 46% men, <2% non-binary). After the piloting was completed (July 2022), we sent our collaborators the final version of the study in Qualtrics, along with an in-depth instructions manual (available at https://osf.io/ujzcx) on how to translate and adapt the study to each country. We also instructed our collaborators to obtain ethics approval from their institutions’ review boards before launching data collection.

It should also be noted that multiple interventions included additional questionnaire items mainly meant to increase participant engagement. These additional items, as well as the number of participants that did and did not respond to these items per condition are available in Supplemental Table S4. These results can be used to help estimate the level of engagement of the participants in the cleaned dataset.

### Survey translations

Consistency of the survey adaptations was ensured in three ways. First, collaborator teams were instructed to use back-translations to ensure that the text was adequate. Should any disputes arise, they were asked to have multiple native speakers work together to help resolve it. Second, teams that were using the same language were strongly encouraged to work together when translating the survey so that they could more evenly distribute the amount of effort that was required. Not only did this help to reduce the likelihood of fatigue by the translators, but it also meant that there were often several native speakers working on the same translations, ensuring that there was a consensus among them. Finally, the organisational team carefully combed through the submitted survey files using different translation software (e.g., DeepL) to ensure that the entire survey had been translated and adapted sufficiently.

### Participants

The data were collected between July 2022 and July 2023. To be included in the cleaned dataset, participants had to be between 18–100 years old, pass two attention checks (i.e., Please select the colour “purple” from the list below.” and “To indicate you are reading this paragraph, please type the word sixty in the text box below.”; the dropout rates by collaborator team are shown in Supplemental Table S6), and pass the WEPT demonstration page. By removing participants who did not pass the attention check, we operated under the assumption that the treatment effects are consistent across both attentive and less attentive groups. This decision was made to enhance data quality while maintaining the assumption of minimal heterogeneous treatment effects.

We also screened the survey files that were uploaded by collaborators to ensure that all translations and country-level adaptations were successfully adopted, and if not, those participants were removed (see the Data Cleaning section). As the main aim of the present data paper is to provide the fullest dataset possible, we opted to include only the above-mentioned inclusion/exclusion criteria when cleaning the data. This allows users to set their own judgements for the boundaries/cutoffs inside of their analyses that make sense for what they would like to do. Thus, there is a small portion of participants included who did not finish the entire survey (2.99% of participants have a 0 in the “Finished” column of the dataset), or participants who did not respond to all items in each subscale.

**Fig. 3:**
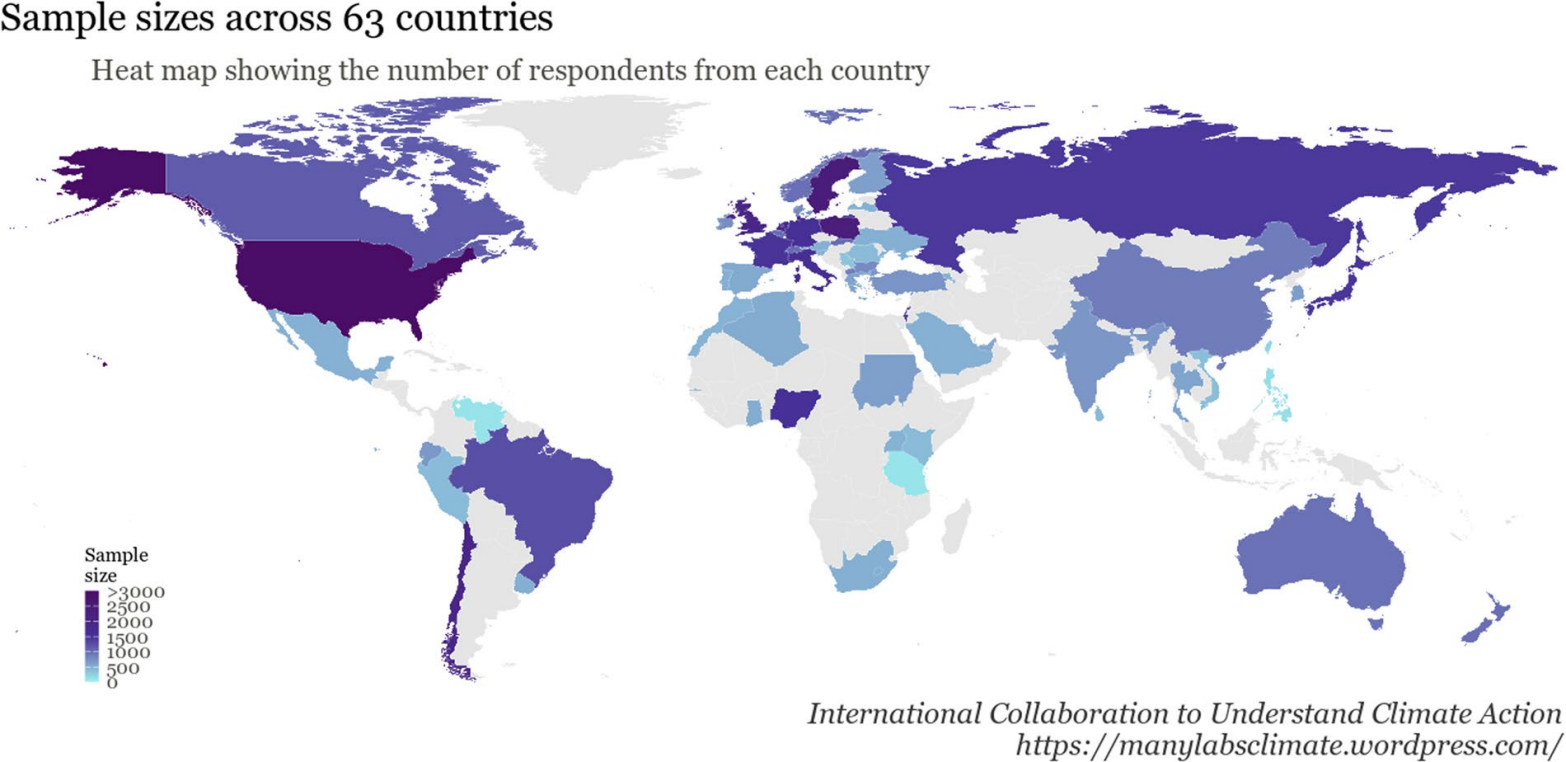
Data distributions. The number of participants in each of the 63 countries represented in the sample (N_total_ = 59,508).

A total of 83,927 people participated, and 59,508 participants (M_age_ = 39.12, SD_age_ = 15.77; 51% women, 47% men, 0.6% non-binary; Figure 3) from 63 countries passed both attention checks and correctly completed the WEPT demonstration. All collaborator team-level descriptive data for age and gender is shown in Supplemental Table S1. Table 1 shows the breakdown for the number of participants that were originally assigned to each group (i.e., “Raw N”) and the number of participants that were included in each condition in the final cleaned dataset (i.e., “Cleaned N”). These values can be used to calculate and adjust for attrition rates across the dataset.

Overall, 75.05% of the entire sample was matched to the population in some way (e.g., census matched regarding age), and 66% of the sample was matched for both age and gender (see Table 2 for the breakdown of all matched variables). Ethics approval was obtained independently by each data collection team from their corresponding Institutional Review Board (IRB). Only datasets submitted, along with IRB approval or an ethics waiver from IRB, are included in the repository.

**Table 2:** Variables on which the samples in each country were matched to the population.

Sample	Matched Variables	N	%	Sample	Matched Variables	N	%
Algeria	N/A	528	0.89	Philippines	N/A	145	0.24
Armenia	N/A	492	0.83	Poland_1	Age, Gender, Education	1883	3.17
Australia	Age, Gender	979	1.65	Poland_2	N/A	463	0.78
Austria	Age, Gender	502	0.84	Portugal	N/A	499	0.84
Belgium_1	Age, Gender	522	0.88	Romania	N/A	411	0.69
Belgium_2	Age, Gender	512	0.86	Russia_1	N/A	718	1.21
Brazil	Age, Gender, Education	1261	2.12	Russia_2	Region, Ethnicity	395	0.66
Bulgaria	Age, Gender	778	1.31	Russia_3	N/A	322	0.54
Canada_1	N/A	858	1.44	Saudi Arabia	N/A	489	0.82
Canada_2	Age, Gender	303	0.51	Serbia	N/A	337	0.57
Chile	Age, Gender, Region, SES	1992	3.35	Singapore	N/A	500	0.84
China	N/A	896	1.51	Slovakia	Age, Gender, Region, Municipality Size	1027	1.73
Czechia	N/A	547	0.92	Slovenia	Age, Gender	501	0.84
Denmark	Age, Gender, Region	792	1.33	South Africa	Age, Gender	496	0.83
Ecuador	Age, Gender, Region	679	1.14	South Korea	Age, Gender	639	1.08
Finland	Age, Gender	625	1.05	Spain_1	N/A	110	0.19
France	Age, Gender	1480	2.49	Spain_2	Age, Gender, Region	434	0.73
Gambia	N/A	527	0.89	Sri Lanka	N/A	413	0.69
Germany	Age, Gender, Region	1545	2.6	Sudan	Age, Gender	623	1.05
Ghana	Age, Gender	522	0.88	Sweden	Age, Gender	2393	4.03
Greece	Age, Gender	597	1	Switzerland_1	Age, Gender	512	0.86
India	N/A	688	1.16	Switzerland_2	Age, Gender	531	0.89
Ireland	N/A	753	1.27	Taiwan	N/A	206	0.35
Israel	Age, Gender, Region, Ethnicity	1384	2.33	Tanzania	Age, Gender	104	0.17
Italy_1	Age, Gender, Region	591	0.99	Thailand	N/A	586	0.99
Italy_2	Gender	993	1.67	Turkey_1	N/A	359	0.6
Japan_1	N/A	653	1.1	Turkey_2	Age, Gender	347	0.58
Japan_2	Income, Education, Region, Ethnicity	802	1.35	Uganda	Age, Gender	476	0.8
Kenya	Age, Gender	409	0.69	UK_1	N/A	235	0.37
Latvia	Income, Education, Ethnicity	485	0.82	UK_2	Age, Gender	952	1.6
Mexico	Age, Gender	490	0.82	UK_3	N/A	287	0.39
Morocco	Age, Gender	474	0.8	UK_4	Gender	501	0.84
Netherlands_1	Age, Gender	854	1.44	Ukraine	N/A	496	0.83
Netherlands_2	Age, Gender	510	0.86	UAE	Broadly representative for age, gender, and nationality^a^	554	0.93
Netherlands_3	N/A	500	0.84	Uruguay	N/A	497	0.84
New Zealand	Gender	1005	1.69	USA_1	Age, Gender	838	1.41
Nigeria	Age, Gender	1513	2.55	USA_2	Age, Gender, Region, Ethnicity	2360	3.97
N. Macedonia	N/A	878	1.48	USA_3	Age, Gender	5055	8.5
Norway	Age, Gender, Ethnicity	997	1.68	Venezuela	N/A	110	0.19
Peru	Age, Gender	405	0.68	Vietnam	N/A	383	0.64

^a^The UAE has a widely diverse and distinctive demographic composition characterized by a significant proportion of expatriate residents as opposed to citizens, and availability of current figures is limited by the infrequent publication of such data. Thus, the data included here is broadly representative.

Regarding the heterogeneity in the dataset, there are several things to note. First, the sampling procedures differed between countries (e.g., the U.S. samples were all census matched on age, and gender while the Slovakian sample was matched on age, gender, region, and municipality size; Table 2). Thus, there is a large amount of heterogeneity within the dataset. Second, while having a sample that is broadly representative of key demographics is ideal, recent work has found that representative samples are not necessarily required to obtain generalisable estimates of effect sizes within countries^23^. Various analyses have highlighted that convenience samples are adequate for estimating treatment effects^23–25^. Thus, the data included in this manuscript should also be suitable, especially for researchers interested in analysing the treatment effects within our sample.

### Experimental design

A dedicated schematic representation of the design can be found in Figure 1. Briefly, all participants were first required to read and acknowledge the informed consent page. At the end of the consent page, participants were exposed to the first attention check (“Please select the color “purple” from the list below. We would like to make sure that you are reading these questions carefully.”). They were then randomly assigned to one of 12 conditions, including the 11 intervention groups (Table 1) or a no-intervention control condition. Participants in the control condition were then exposed to a short, thematically unrelated text from the novel “Great Expectations” by Charles Dickens in order to balance the amount of time spent on this phase of the experiment. Next, all participants were exposed to a definition of climate change: “Climate change is the phenomenon describing the fact that the world’s average temperature has been increasing over the past 150 years and will likely be increasing more in the future.” Participants in the intervention groups were then exposed to their intervention.

All participants were then directed to the dependent variable phase, where, in random order, they rated their (1) climate beliefs, (2) climate policy support, and (3) were given the option to create a social media post. Finally, they could contribute to the tree-planting effort by completing the WEPT. Note that the WEPT was always the last outcome variable measured, while the other three outcomes were measured randomly. Next, participants in the control condition were asked to complete a series of additional variables (described below). Finally, participants were asked to report their demographic information, which included another attention check (“In the previous section, you viewed some information about climate change. To indicate you are reading this paragraph, please type the word sixty in the text box below.”).

### Primary Outcomes

**Fig. 4:**
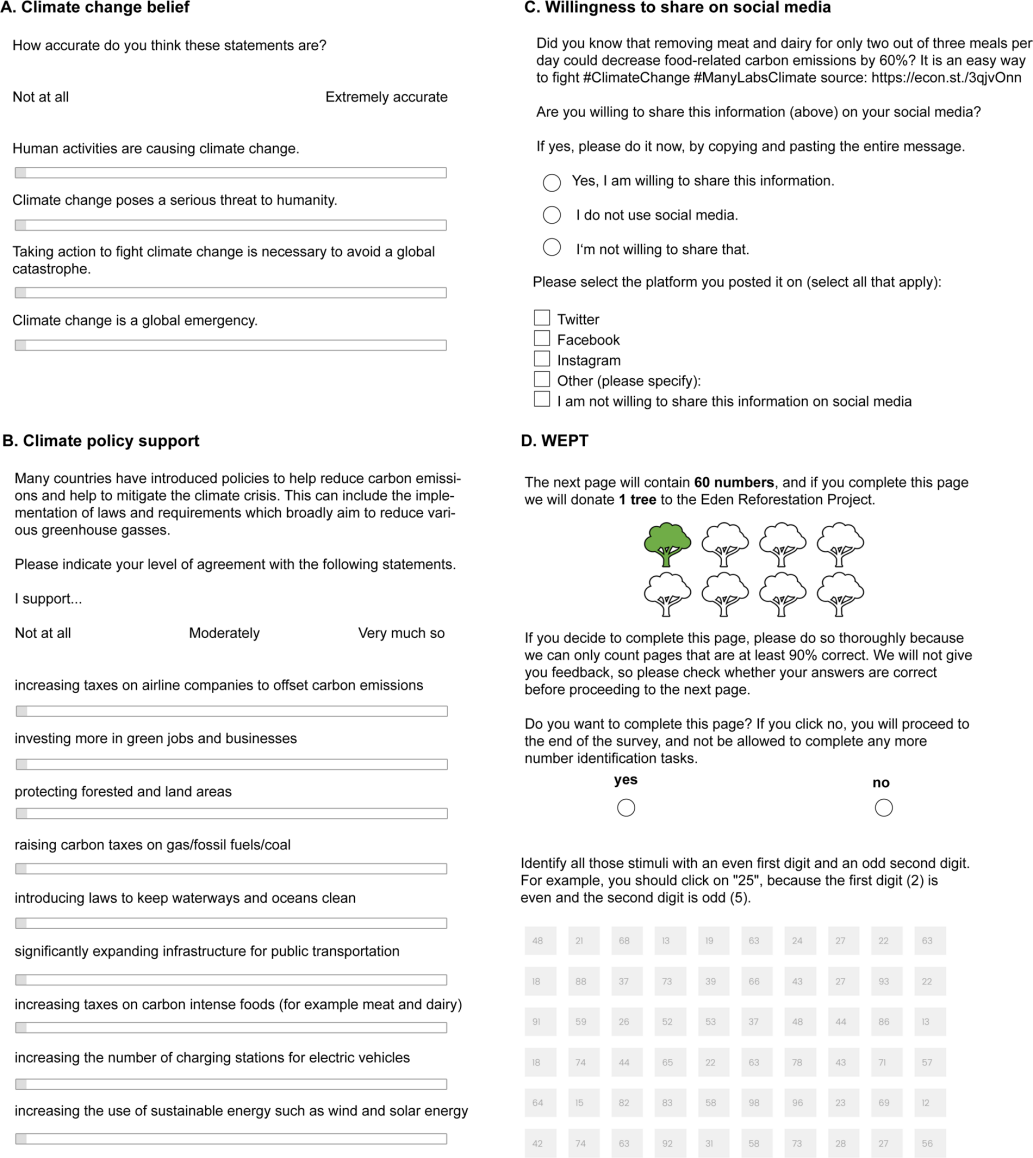
Graphic illustration of the primary outcome variables. (**A**) climate change belief, (**B**) climate policy support, (**C**) willingness to share on social media, (**D**) the WEPT.

Figure 4 shows graphic illustrations of the four primary outcome variables.

#### Climate change beliefs

Climate beliefs were measured by participants’ answers to the question “How accurate do you think these statements are?” from *0* = *Not at all accurate to* 100 = *Extremely accurat*e. The four statements were: “Taking action to fight climate change is necessary to avoid a global catastrophe,” “Human activities are causing climate change,” “Climate change poses a serious threat to humanity,” and “Climate change is a global emergency.”

#### Climate change policy support

This dependent variable consisted of participants’ level of agreement from *0* = *Not at all* to *100* = *Very much so* using a slider (participants could also respond with “not applicable”, which is coded as “NA” in the dataset), with the following nine statements: “I support raising carbon taxes on gas/fossil fuels/coal,” “I support significantly expanding infrastructure for public transportation,” “I support increasing the number of charging stations for electric vehicles,” “I support increasing the use of sustainable energy such as wind and solar energy,” “I support increasing taxes on airline companies to offset carbon emissions,” “I support protecting forested and land areas,” “I support investing more in green jobs and businesses,” “I support introducing laws to keep waterways and oceans clean,” and “I support increasing taxes on carbon-intensive foods (for example, meat and dairy).”

#### Willingness to share climate information on social media

Participants were first presented with the text, “Did you know that removing meat and dairy for only two out of three meals per day could decrease food-related carbon emissions by 60%? It is an easy way to fight #ClimateChange #ManyLabsClimate${e://Field/cond} source: https://econ.st/3qjvOnn” (where “{e://Field/cond}” was replaced with the condition code for each group; an example can be found here https://bit.ly/3FKcwyq). Participants were then asked, “Are you willing to share this information on your social media?” the answer options were “Yes, I am willing to share this information,” “I am not willing to share this information,” and “I do not use social media.” Participants who indicated they do not use social media (N = 15,252, 25.9% of the sample) were recoded as NA in this variable to avoid confusion and to exclude them from relevant analyses. Moreover, participants were asked to indicate the platform (e.g., Facebook, Twitter, Instagram) on which they posted the information.

#### WEPT Tree planting efforts

We used a modified version of the Work for Environmental Protection Task (WEPT) to measure an action with a real-world impact performed at an actual cost to participants^14^. This task is a multi-trial web-based procedure that detects consequential pro-environmental behaviour by allowing participants the opportunity to engage in voluntary cognitive effort (i.e., screening numerical stimuli) in exchange for donations to an environmental organisation. This measure has been validated and has been found to correlate to self-reports and objective observations of other pro-environmental behaviours and conceptually related measures^14,26^.

Participants were first exposed to a demonstration of the WEPT, in which they were instructed to identify all target numbers for which the first digit is even and the second digit is odd (4 out of 18 numbers were target numbers on the demonstration page). Participants could not advance the page until they correctly completed the WEPT demonstration. They were then told that planting trees is one of the best ways to combat climate change and that they would have the opportunity to plant up to 8 trees if they chose to engage in additional pages of the item identification task (one tree per page of WEPT completed). These pages contained 60 numbers per page, which participants had to screen for target numbers. Alongside these instructions, participants were shown a pictogram of 8 trees, one of which was coloured green to mark their progress in the task (Figure 4D). Participants were allowed to exit the task at any point with no penalty.

Due to the participants’ efforts, 333,333 trees were planted in collaboration with The Eden Reforestation Project. Assuming that the average fully-grown tree absorbs between 10 and 40 kg of carbon dioxide per year, in 5–10 years when all trees are fully grown, the efforts from this project will result in approximately 9,999,990 kg of carbon dioxide sequestered per year, which is the equivalent amount of carbon dioxide used to produce energy for 1,260 US homes per year.

### Additional independent variables

As shown in Figure 1, participants from the no-intervention control condition were also required to complete a set of additional independent variables. The items included are listed in Supplemental Table S3.

### Demographic block

**Fig. 5:**
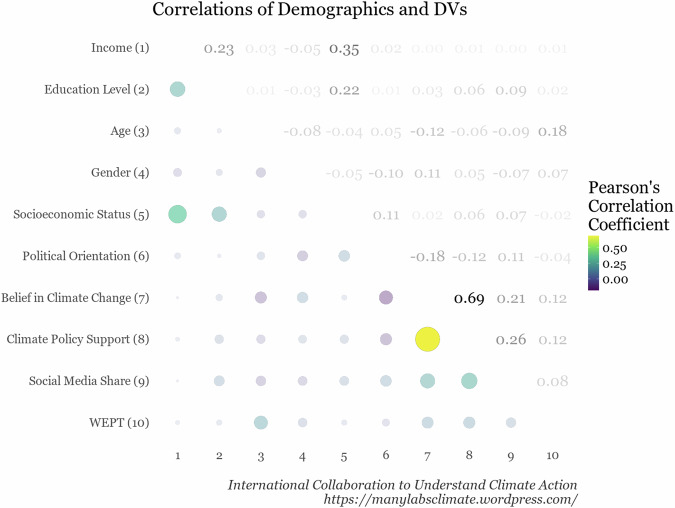
Correlation matrix showing the Pearson’s correlations between the demographic predictors and the four outcome variables.

After briefly explaining why we were measuring some background information, we then measured a series of demographic variables (see Supplemental Table S5). The correlation plot between the variables from the demographic block and the primary outcomes is shown in Figure 5.

### Data cleaning

We received individual data files from each collaboration team in either .csv or .xlxs format as well as the Qualtrics files (i.e.,.qsf) from the survey (information about each data submission can be found at: https://osf.io/sd5qb). Each team’s survey file was visually inspected by at least two members of the organisational team (mainly BT & PS) to ensure that they were adapted and translated fully. While some interventions required only translation, others (Work Together Norm, System Justification, Psychological Distance, Pluralistic Ignorance, Dynamic Social Norms, Binding Moral Foundation) required further adaptations on a country-level (the collaborator manual outlining all adaptations can be found at https://osf.io/ujzcx). For example, the Binding Moral Foundation intervention contained an image of a person holding a flag, thus, a different image with the respective flag for the country was required. If the image was not changed, we removed the participants receiving this intervention from that collaborator team’s data. We documented all unsuccessful/partial translations and adaptation of the interventions (see https://osf.io/wu6gf for an overview).

The measure for socioeconomic status contained the respective country name, so we inspected the surveys and documented if the name has not been changed to reflect the correct country (see https://osf.io/ueqgy for an overview). Additionally, we documented which teams had changed the coding of some of the variables (see https://osf.io/qbe84 for gender, https://osf.io/5ypca for education). Before we merged the individual datasets, we changed the data from the participants who did not give consent to NAs. To merge and clean these raw data, minor modifications were introduced, which are briefly described below, and fully documented in the dataset merging script (https://osf.io/uam3y) and cleaning script (https://osf.io/4rm7g).

In the merging script, each dataset was imported into R individually. When encountering ambiguous date formats (such as those found in start date, end date, and record date), we manually specified the correct format and standardized them. Column names which were inconsistent with the original survey were renamed or removed, and the attention checks were recorded to ensure accuracy. The merged raw dataset can be found on OSF (see https://osf.io/snuwd).

In the cleaning script, all variables were checked to ensure they were coded in a consistent and comparable way. For example, there were some mistakes with the way that education was coded for some teams, so the data were individually recoded. Next, the empty rows for the non-consenting participants were removed, as well as survey tests that some teams did not remove when submitting their data. Next, participants who were not assigned a condition due to technical issues were removed (N = 1,753), as well as participants with invalid age values (less than 18 or more than 100, N = 157). Any errors that were identified for the survey translation and adaptation were then corrected individually, and participants were removed accordingly (N = 1,010). Participants who did not pass the two attention checks (first: N = 574, second: N = 20,194), nor the WEPT demonstration (N = 354) were then removed. The cleaned dataset can be found on OSF (see https://osf.io/xum6b).

## Data Records

All materials for this project are openly available on the project’s repository hosted on Open Science Framework (https://osf.io/ytf89/)^13^.

### Navigating the repository

The file repository is organised in several folders:

- **ClimateManylabs_Code** folder contains R scripts, including the code for merging the raw datasets submitted by each of the collaborators (datapaper_merging_raw.R), the code for cleaning the data (datapaper_cleaning.R) and the code for reproducing the figures (datapaper_figures_code.R).
- **ClimateManylabs_CollaboratorResources** contains the document with the information on ethics application (ethics_application_materials.pdf), the manual the collaborators received for adapting the interventions to their country and language (intervention_adaptation_manual.pdf) and a pdf file containing the master survey items (master_survey.pdf).
- **ClimateManylabs_Data** contains the single raw data files (i.e. all of the submitted datasets from all of the collaborators in a compressed form - countries_rawdata.7z), the merged raw dataset (data_raw.csv), the cleaned dataset (data_countries.csv), an additional cleaned version without the timers (data_notimers.csv), a codebook for navigating the dataset (codebook.xlsx), the items of the survey we used when asking the collaborators to submit their datasets (data_submission_survey.pdf), and the responses to this survey (manylabsclimate_datasubmission.csv).
- **ClimateManylabs_InterventionTournamentVote** contains the Qualtrics survey file (intervention_vote_manylabs.qsf) used for evaluating the interventions, the data of this survey (vote_data.xlsx), and the pdf file where the items of the survey can be seen (tournament_survey_items.pdf).
- **ClimateManylabs_IRBs** contains all of the approvals by the ethics boards in the different institutions.
- **ClimateManylabs_QSF** contains all the Qualtrics survey files (.qsf) that the collaborators used to collect their data.
- **ClimateManylabs_Supplementary** contains a supplementary figure with the data collection dates (data_collection_dates.png), an overview table of how education was coded (education_coding_overview.xlsx), how gender was coded (gender_coding_overview.xlsx), an overview of whether the interventions were translated and adapted correctly (intervention_translation_and_adaptation_overview.xlsx), a table containing the internal consistencies of the measures used in the survey, calculated per country (measures_internal_consistency_per_country.csv), and an overview of the adaptation of the socioeconomic status ladder per country (SES_ladder_countryname_adaptation_overview.xlsx).

An easy to access guide on navigating the repository can be found in the READme.txt file on the OSF platform.

## Technical Validation

**Fig. 6:**
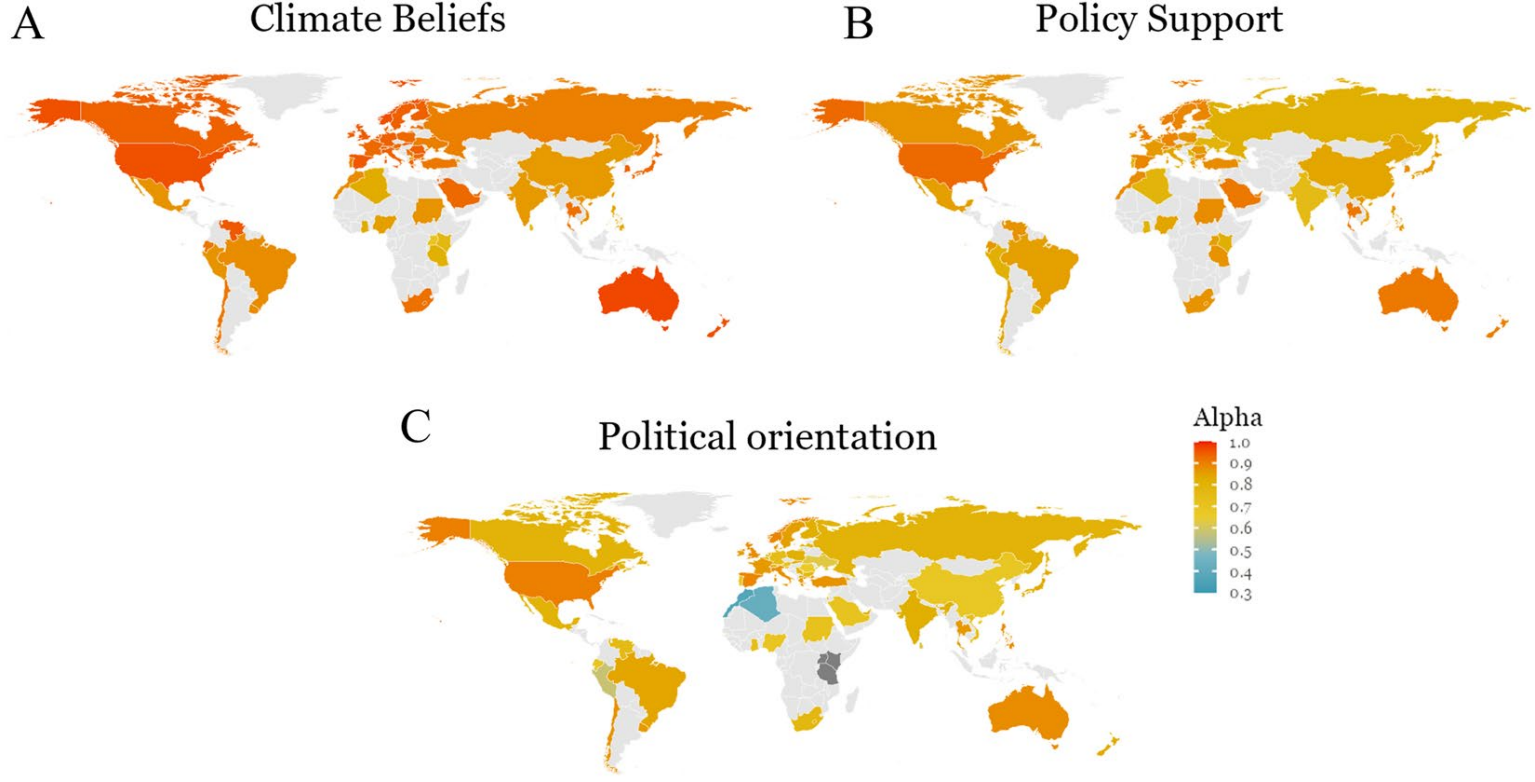
Internal consistency (Cronbach’s alpha) of the items measuring (**A**) climate change beliefs, (**B**) policy support, and (**C**) political orientation, calculated and plotted separately for each country.

Similar to a previously published many labs dataset^27^, we calculated numerous indicators of internal consistency at the country level for any scale (Table 2, Supplemental Tables S4-5) that contained more than two items. This included Cronbach’s Alpha, McDonald’s Omega, Guttman’s split-half reliability, and the proportion of variance explained by a unidimensional factor. The average of these measures is shown in Table 3. The full table of results can be found at https://osf.io/ejtdq, and visualisations of Cronbach’s alpha for climate belief, policy support, and political orientation are shown in Figure 6. Visualisations of Cronbach’s alpha for all other variables from Table 3 are shown in Supplemental Figure S2. Across these reliability measures, the majority of variables had good (Cronbach’s alpha > 0.70) to excellent (Cronbach’s alpha > 0.80) internal consistency.

**Table 3:** Average and standard deviations (brackets) for the reliability measures averaged across all 63 countries.

Measure	Cronbach’s Alpha	Guttman’s split-half coefficient	McDonald’s Omega	Proportion of variance explained
Climate Belief	0.90 (0.06)	0.91 (0.05)	0.90 (0.05)	0.71 (0.11)
Policy Support	0.86 (0.04)	0.90 (0.03)	0.85 (0.05)	0.42 (0.07)
Political Orientation	0.77 (0.10)	0.77 (0.10)	0.77 (0.10)	0.64 (0.13)
Environmental Identity	0.90 (0.05)	0.93 (0.04)	0.90 (0.04)	0.70 (0.10)
External Motivation	0.85 (0.07)	0.86 (0.04)	0.86 (0.06)	0.58 (0.10)
Internal Motivation	0.70 (0.17)	0.77 (0.09)	0.78 (0.08)	0.47 (0.10)
Trust in Climate Science	0.85 (0.13)	0.85 (0.13)	0.85 (0.12)	0.75 (0.15)

## Usage Notes

We recommend using one of the cleaned datasets. One dataset, which includes all participant timers, and number of clicks per page can be found at https://osf.io/xum6b, and a version without any timers/click counts can be found at: https://osf.io/8q6ue. For more information on how to navigate the OSF repository read the uploaded READme.txt file (https://osf.io/8wh9m).

## Supplementary information

Supplementary Information

## Data Availability

All data (raw and cleaned), the materials from the study (e.g., Qualtrics surveys, IRB forms, etc.), codebooks, and the code presented in this manuscript are available at https://osf.io/ytf89.
