# Supplementary material for: The International Climate Psychology Collaboration: Climate change-related data collected from 63 countries
Source: Sci Data. 2024 Oct 1;11:1066. doi: 10.1038/s41597-024-03865-1 (PMC11445540; doi:10.1038/s41597-024-03865-1)
Supplement: Supplementary file 1 — Supplementary Information [file 41597_2024_3865_MOESM1_ESM.docx]

**Supplemental Information**

**Supplemental Figures**


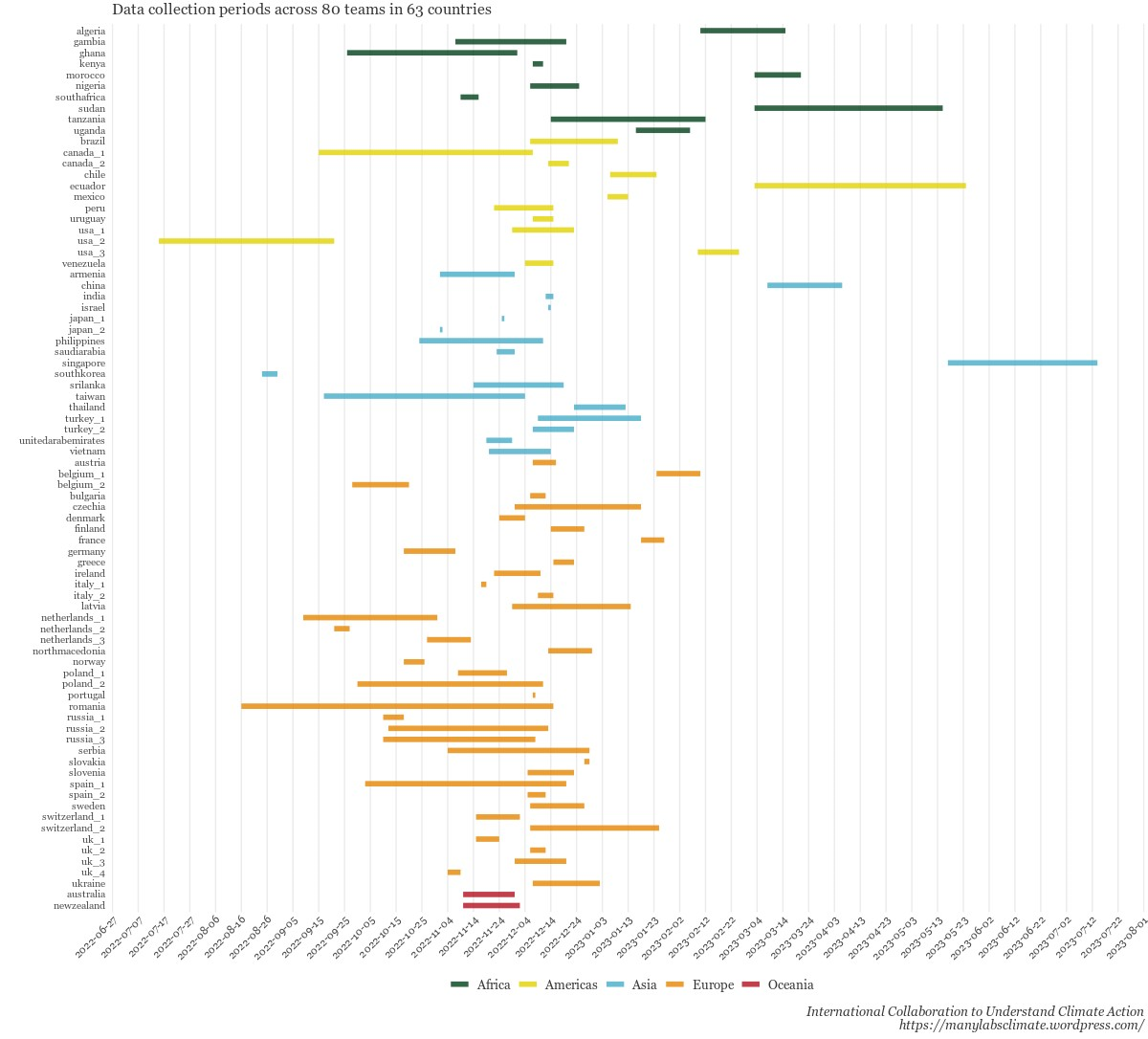


**Supplemental Figure S1.** **Gantt Chart illustrating the data collection periods for each surveyed country**. A high-resolution version of this image can be found at <https://osf.io/tpw2c>.


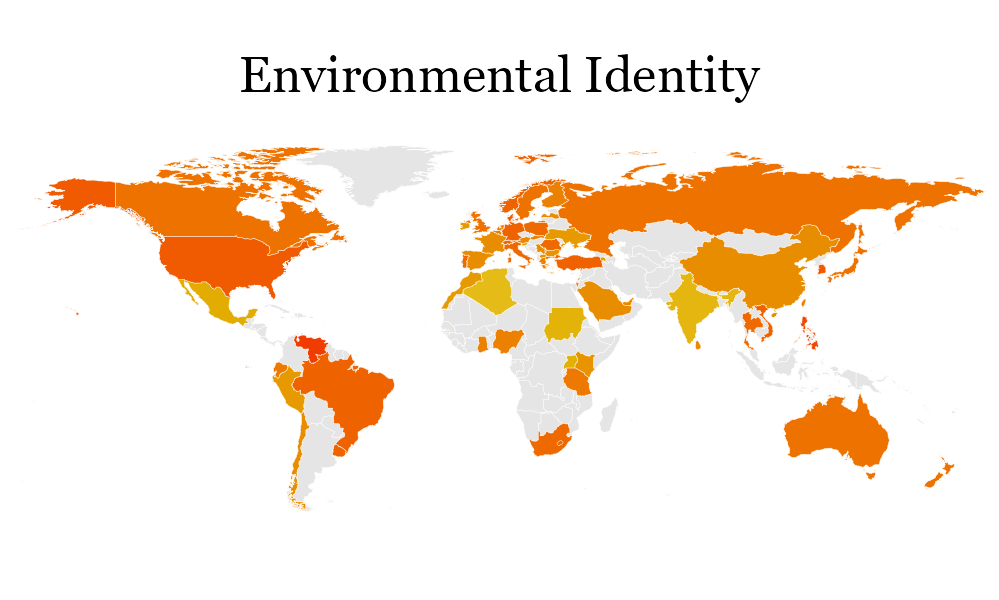

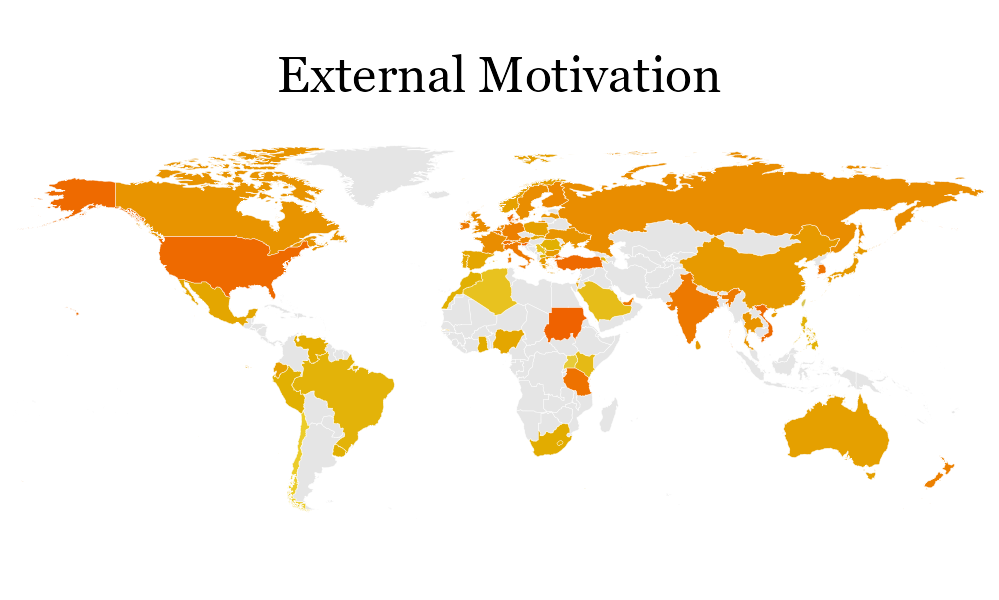

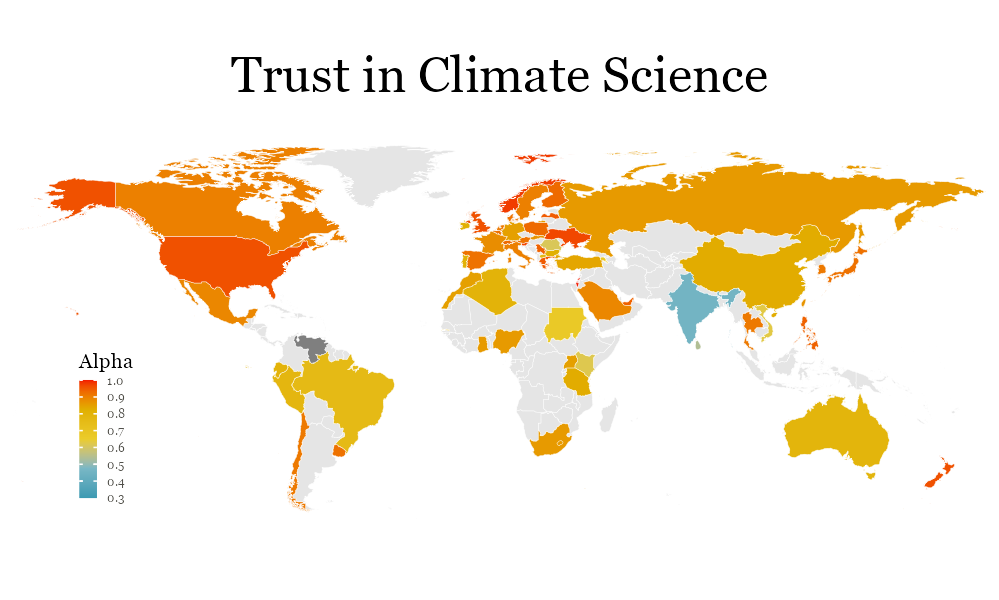


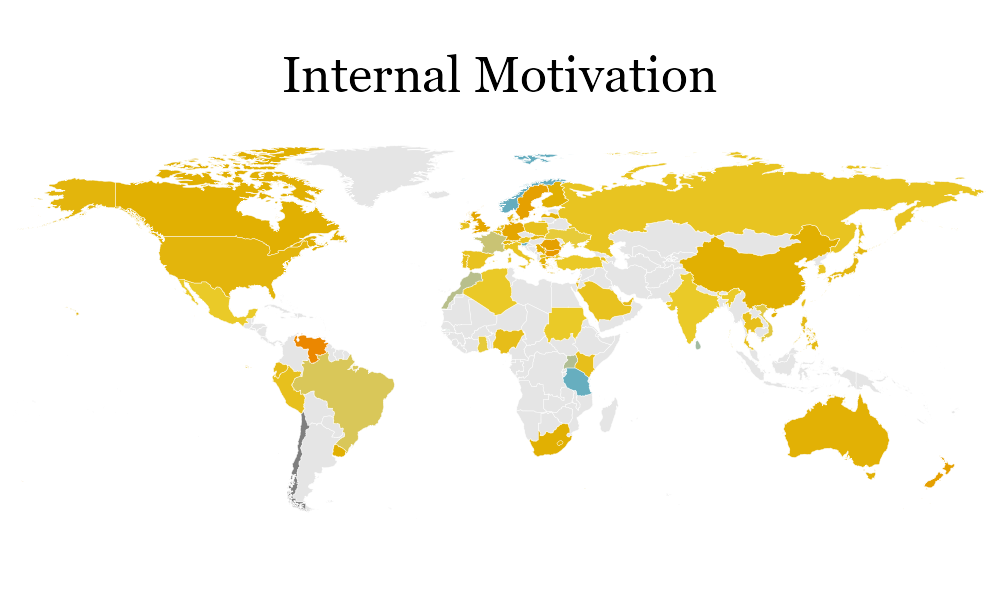

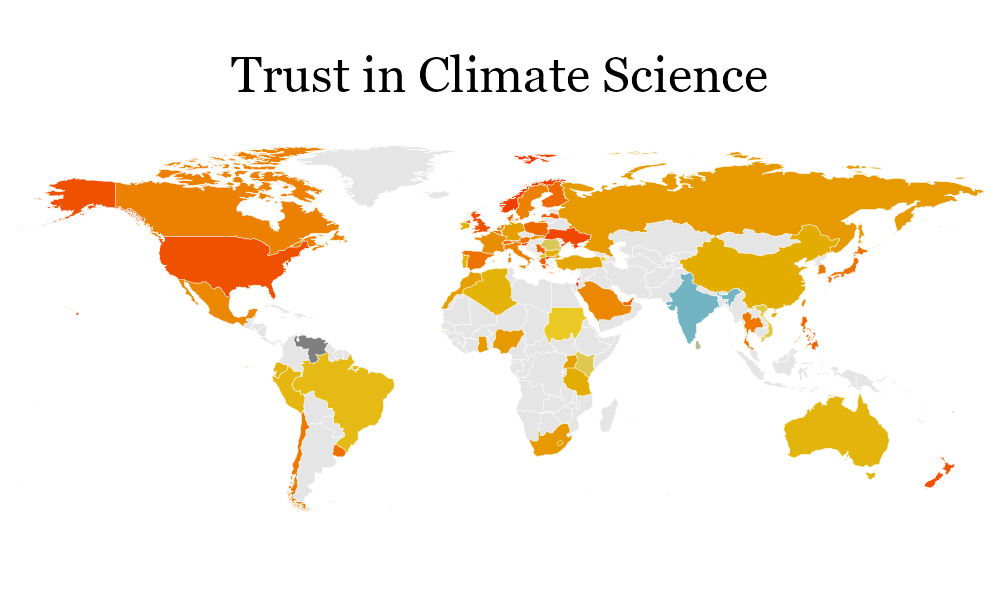


Supplemental Figure S2. Cronbach’s alpha reliability score in 63 countries.

**Supplemental Tables**

| **Supplemental Table S1**: Descriptives of the age, and gender makeup of participants included across all data collection teams. | | | | | | | | |
| --- | --- | --- | --- | --- | --- | --- | --- | --- |
| **Team** | **N** | **Age (Mean)** | **Age (SD)** | **Age percentage of responses** | **Gender percentage of males** | **Percentage of females** | **Percentage of non-binary/other** | **Percentage of NA/prefer not to say** |
| Algeria | 528 | 32.3 | 9.5 | 99.6 | 69.7 | 29.7 | 0 | 0.6 |
| Armenia | 492 | 32 | 13.8 | 99.8 | 36 | 63.4 | 0.2 | 0.4 |
| Australia | 979 | 46.1 | 15.7 | 100 | 47.1 | 52.7 | 0.2 | 0 |
| Austria | 502 | 37.9 | 13.4 | 100 | 49.2 | 50 | 0.6 | 0.2 |
| Belgium_1 | 522 | 48.7 | 16.5 | 93.1 | 48.3 | 44.1 | 0.8 | 6.9 |
| Belgium_2 | 512 | 45.9 | 14.6 | 99.6 | 49.6 | 49.8 | 0.2 | 0.4 |
| Brazil | 1261 | 38.1 | 13.8 | 99.4 | 48.3 | 51.3 | 0.3 | 0.1 |
| Bulgaria | 778 | 41.3 | 13.4 | 99.5 | 50.3 | 49 | 0.1 | 0.6 |
| Canada_1 | 858 | 20.5 | 3.5 | 99.9 | 24.2 | 72 | 2.2 | 1.5 |
| Canada_2 | 303 | 46.3 | 15.3 | 100 | 49.5 | 49.8 | 0.3 | 0.3 |
| Chile | 1992 | 42.3 | 18 | 22.4 | 13.1 | 9.2 | 0.3 | 77.4 |
| China | 896 | 28.3 | 7.2 | 100 | 38.2 | 61.5 | 0.1 | 0.2 |
| Czechia | 547 | 26.1 | 11.4 | 100 | 30.3 | 67.5 | 1.6 | 0.5 |
| Denmark | 792 | 48.5 | 18 | 63.8 | 33.2 | 30.7 | 0 | 36.1 |
| Ecuador | 679 | 33.5 | 10.5 | 99 | 51 | 46.1 | 1 | 1.9 |
| Finland | 625 | 42.9 | 15 | 99.8 | 42.1 | 57.3 | 0.3 | 0.3 |
| France | 1480 | 47.1 | 15.8 | 99.7 | 46.9 | 52.6 | 0.3 | 0.2 |
| The Gambia | 527 | 24.3 | 6.4 | 97.9 | 51.2 | 45.4 | 1.1 | 2.3 |
| Germany | 1545 | 46.9 | 15.8 | 99.7 | 51.7 | 47.3 | 0.2 | 0.8 |
| Ghana | 522 | 29.1 | 8.6 | 99.6 | 58 | 39.5 | 1 | 1.5 |
| Greece | 597 | 39.4 | 12.2 | 98.2 | 55.3 | 43.2 | 0.3 | 1.2 |
| India | 688 | 26.8 | 8.5 | 100 | 62.9 | 34.7 | 1 | 1.3 |
| Ireland | 753 | 27.2 | 9.3 | 99.5 | 48.9 | 48.9 | 0.8 | 1.5 |
| Israel | 1384 | 38.6 | 12.9 | 100 | 51.4 | 48 | 0.2 | 0.4 |
| Italy_1 | 591 | 46.9 | 14.8 | 84.8 | 42 | 42.8 | 0 | 15.2 |
| Italy_2 | 993 | 29.7 | 9.2 | 100 | 50.1 | 47.5 | 2.1 | 0.3 |
| Japan_1 | 653 | 42.1 | 10.2 | 100 | 54.7 | 45.2 | 0 | 0.2 |
| Japan_2 | 802 | 48.9 | 11.5 | 100 | 70.7 | 27.4 | 0.2 | 1.6 |
| Kenya | 409 | 31.8 | 13.6 | 100 | 56.2 | 43.3 | 0.2 | 0.2 |
| Latvia | 485 | 51.6 | 15.2 | 99.4 | 26.2 | 72 | 0.8 | 1 |
| Mexico | 490 | 38.6 | 13.3 | 100 | 49.2 | 50.2 | 0.6 | 0 |
| Morocco | 474 | 34.2 | 11.7 | 99.4 | 56.5 | 42.8 | 0 | 0.6 |
| Netherlands_1 | 854 | 30.2 | 14.8 | 99.9 | 27.4 | 71.5 | 0.7 | 0.4 |
| Netherlands_2 | 510 | 47.6 | 16.5 | 100 | 49.6 | 50 | 0.2 | 0.2 |
| Netherlands_3 | 500 | 47.4 | 16.2 | 100 | 48.2 | 51 | 0.6 | 0.2 |
| New Zealand | 1005 | 46.3 | 16 | 100 | 45.7 | 54.1 | 0.2 | 0 |
| Nigeria | 1513 | 32.1 | 9.7 | 99.4 | 61.7 | 37.5 | 0 | 0.8 |
| North Macedonia | 878 | 26.2 | 11.9 | 99.2 | 28 | 68.3 | 1.3 | 2.4 |
| Norway | 997 | 52.6 | 14.8 | 99.5 | 51.8 | 46.4 | 1 | 0.8 |
| Peru | 405 | 24.2 | 7.7 | 99.8 | 37.5 | 59.5 | 1.5 | 1.5 |
| Philippines | 145 | 27.4 | 7.9 | 98.6 | 29.7 | 62.1 | 2.8 | 5.5 |
| Poland_1 | 1883 | 47.1 | 16.3 | 99.6 | 48.3 | 51 | 0.5 | 0.2 |
| Poland_2 | 463 | 27.7 | 8.6 | 99.6 | 12.7 | 84.9 | 1.9 | 0.4 |
| Portugal | 499 | 27.2 | 7.9 | 100 | 50.1 | 48.5 | 1.4 | 0 |
| Romania | 411 | 42.7 | 14 | 98.8 | 66.7 | 31.9 | 0.2 | 1.2 |
| Russia_1 | 718 | 35.6 | 11.8 | 99.3 | 49.7 | 49 | 0.1 | 1.1 |
| Russia_2 | 395 | 24.9 | 10.1 | 100 | 17.5 | 80.5 | 0.5 | 1.5 |
| Russia_3 | 322 | 22 | 7.2 | 100 | 37.6 | 60.9 | 0.3 | 1.2 |
| Saudi Arabia | 489 | 34.4 | 8.7 | 98 | 54 | 45.8 | 0 | 0.2 |
| Serbia | 337 | 40.4 | 13.5 | 97.6 | 27 | 70 | 0.3 | 2.7 |
| Singapore | 500 | 36.3 | 11.6 | 99.6 | 49 | 50.2 | 0 | 0.8 |
| Slovakia | 1027 | 44.4 | 15.4 | 99.4 | 46.4 | 52.6 | 0.5 | 0.5 |
| Slovenia | 501 | 34.1 | 11.3 | 99.8 | 58.3 | 40.5 | 0.4 | 0.8 |
| South Africa | 496 | 35.9 | 12.3 | 99.2 | 46 | 53.2 | 0 | 0.8 |
| South Korea | 639 | 43.8 | 13.8 | 100 | 48.8 | 50.9 | 0 | 0.3 |
| Spain_1 | 110 | 38.5 | 14.5 | 99.1 | 36.4 | 59.1 | 0.9 | 3.6 |
| Spain_2 | 434 | 46.6 | 15.4 | 99.8 | 49.3 | 48.8 | 0.9 | 0.9 |
| Sri Lanka | 413 | 27.7 | 7.7 | 100 | 73.4 | 23.2 | 1 | 2.4 |
| Sudan | 623 | 31.4 | 7.1 | 99 | 67.9 | 31.3 | 0 | 0.8 |
| Sweden | 2393 | 40.6 | 15.4 | 99.3 | 45.8 | 52.8 | 0.6 | 0.8 |
| Switzerland_1 | 512 | 45.2 | 14.9 | 99.8 | 48.6 | 50.8 | 0 | 0.6 |
| Switzerland_2 | 531 | 43.9 | 14.7 | 99.6 | 49 | 50.5 | 0.2 | 0.4 |
| Taiwan | 206 | 31.1 | 9.6 | 99 | 43.2 | 51.5 | 3.4 | 1.9 |
| Tanzania | 104 | 28.4 | 7.7 | 98.1 | 70.2 | 26 | 1.9 | 1.9 |
| Thailand | 586 | 36.1 | 12.1 | 97.1 | 55.6 | 40.4 | 3.2 | 0.7 |
| Turkey_1 | 359 | 25 | 8.2 | 99.2 | 17 | 81.1 | 0.3 | 1.7 |
| Turkey_2 | 347 | 39.7 | 13.1 | 100 | 47.6 | 51.9 | 0.3 | 0.3 |
| Uganda | 476 | 36.1 | 13.9 | 99.8 | 51.3 | 47.9 | 0.4 | 0.4 |
| UK_1 | 235 | 32.4 | 10.1 | 100 | 46 | 54 | 0 | 0 |
| UK_2 | 952 | 44.4 | 16.1 | 99.8 | 40.8 | 58.5 | 0.3 | 0.4 |
| UK_3 | 287 | 23.7 | 8.2 | 99.7 | 18.8 | 63.1 | 7.7 | 10.5 |
| UK_4 | 501 | 41.3 | 14.8 | 100 | 49.7 | 48.5 | 1.6 | 0.2 |
| Ukraine | 496 | 30.7 | 11.4 | 99.2 | 29.4 | 68.3 | 1 | 1.2 |
| United Arab Emirates | 554 | 34.5 | 8.9 | 98.7 | 52.3 | 47.3 | 0 | 0.4 |
| Uruguay | 497 | 37.4 | 12.3 | 99.8 | 29.6 | 68.6 | 1 | 0.8 |
| USA_1 | 838 | 43.7 | 15.1 | 99.8 | 45.7 | 52.4 | 1.1 | 0.8 |
| USA_2 | 2360 | 45.5 | 16.5 | 99.6 | 48 | 49.5 | 1.9 | 0.6 |
| USA_3 | 5055 | 46.4 | 16.2 | 99.5 | 47.7 | 50.7 | 0.7 | 0.9 |
| Venezuela | 110 | 52.4 | 14.9 | 100 | 60.9 | 35.5 | 2.7 | 0.9 |
| Vietnam | 383 | 22.6 | 7 | 99.5 | 25.3 | 72.8 | 1 | 0.8 |

| **Supplemental Table S2**. Names of ethics review boards and corresponding files for all data collection teams. | | |
| --- | --- | --- |
| **Country team** | **Name of Ethics Review Board** | **Name of file on OSF** |
| Algeria | Aarhus University’s Research Ethics Committee | IRB_Pfattheicher_Algeria.pdf |
| Armenia | RUHR-UNIVERSITÄT BOCHUM Fakultät für Psychologie Ethikkommission | IRB_HOFMANN_Armenia.pdf |
| Australia | Research Ethics Office, The Australian National University | IRB_KLAS_New Zealand |
| Austria | The Ethics Committee of the Faculty of Business, Economics and Social Sciences  of the University of Bern | IRB_BERGER_Switzerland |
| Belgium 1 | Comité d’Avis Ethique de la Faculté des Sciences Psychologiques et de l'Education, Université Libre de Bruxelles | IRB_BERTIN_French-speaking-Belgium |
| Belgium_2 | Sociaal-Maatschappelijke Ethische Commissie (SMEC), KU Leuven | IRB_LANGE_Belgium |
| Brazil | COMISSÃO NACIONAL DE ÉTICA EM PESQUISA | IRB_REGO_Brazil |
| Bulgaria | The Ethics Committee of the Faculty of Business, Economics and Social Sciences  of the University of Bern | IRB_BERGER_Switzerland |
| Canada_1 | The University of British Columbia, Office of Research Services, Behavioural Research Ethics Board | IRB_LUO_Canada |
| Canada_2 | Simon Frazer University Research Ethics | IRB_LUTZ_Canada |
| Chechia | IRB, Department of Management, Prague University of Economics and Business | IRB_SAY_Czechia |
| Chile | Comité Ético Científico de Ciencias Sociales, Pontificia Universidad Católica de Chile | IRB_Carvacho_Chile |
| China (TW) | Northwestern University, Institutional Review Board | IRB_Zhao_China |
| Denmark | The Research Ethics Commitee, Aarhus BSS | IRB_Elbaek_Denmark |
| Ecuador | University of Oslo, Faculty of Social Sciences, Department of Psychology’s Research Ethics Committee | IRB_Haugestad_Ecuador |
| Finland | IRB Universitetet i Stavanger, Det samfunnsvitenskapelige fakultet/ Institutt for sosialfag | IRB_GRASSINI_Finland |
| France | CUREG, Université de Genéve | IRB_BROSCH_France |
| Gambia | Ethikkommission Paris-Lodron-Universität Salzburg | IRB_Farage_The Gambia |
| Germany | The Ethics Committee of the Faculty of Business, Economics and Social Sciences  of the University of Bern | IRB_BERGER_Switzerland |
| Ghana | Ethikkommission der Fakultät für Mathematik, Informatik und Statistik, LMU München | IRB_Feuerriegel_Ghana |
| Greece | Univeristy of Crete Research Ethics Committee | IRB_Gkinopoulos_greece |
| India | Ethics Review Board (FMG-UvA), University of Amsterdam | IRB_Dubey_India |
| Ireland | EHS Research Ethics, University of Limerick | IRB_Griffin_Ireland |
| Israel | Social Sciences Ethics Committee, The Hebrew Univesristy of Jerusalem | IRB_Cohen-EICK_Israel |
| Italy_1 | Institutional Review Board for Social & Behavioral Sciences, University of Virginia | IRB_Cian_italy |
| Italy_2 | COMITATO ETICO DELLA RICERCA PSICOLOGICA, Dipartimenti/Sezione di Psicologia, Università di Padova | IRB_Tedaldi_Italy |
| Japan_1 | IRB of the Kochi University of Technology | IRB_HIMICHI_Japan.pdf |
| Japan_2 | The Ethics Review Committee on Research with Human Subjects of  Waseda University | IRB_ISHII_Japan.pdf |
| Kenya | The Science-Geosciences Ethics Review Board (SG ERB) at Utrecht University | IRB_VAN DEN BROEK_KenyaTanzaniaUganda.pdf |
| Latvia | The IRB of the Latvijas Universitātes Humanitāro | IRB_Vanags_Latvia.pdf |
| Mexico | Research Commission of the Leuphana University of Lüneburg | IRB_LOSCHELDER_Mexico.pdf |
| Morocco | Institutional Review Board at NYU Abu Dhabi | IRB_Bélanger_Morocco_Sudan.pdf |
| Netherlands_1 | Ethics Review Board, Tillburg School of Social and Behavioral Sciences, Tilburg University | IRB_vanSchie_Netherlands.pdf |
| Netherlands_2 | Ethics Review Board, Communication Science, University of Amsterdam | IRB_Scholz_Netherlands.pdf |
| Netherlands_3 | Ethics Review Board, Communication Science, University of Amsterdam | IRB_Scholz_Netherlands.pdf |
| New Zealand | the Science & Med DERC Chair at the Australian National University | IRB_Klas_New_Zealand.pdf |
| Nigeria | Science, Technology, Engineering, and Mathematics Ethical Review Committee at the University of Birmingham | IRB_CUTLER_Nigeria.pdf |
| North Macedonia | Етичкиот поткомитет за медицина, фармација, ветерина и стоматологија при МАНУ, Македонска академија на науките и уметностите | IRB_GJONESKA_Macedonia.pdf |
| Norway | Norwegian School of Economics Institutional Review Board | IRB_Sjaastad_Norway.pdf |
| Peru | Universidad Peruana Cayetano Heredia | IRB_MONGE_PERU.pdf |
| Philippines | University of the Philippines Visayas RESEARCH ETHICS BOARD | IRB_Guilaran_Philippines.pdf |
| Poland_1 | Faculty of Philosophy and Social Sciences, Nicolaus Copernicus University | IRB_Herman_poland.pdf |
| Poland_2 | Faculty of Philosophy and Social Sciences, Nicolaus Copernicus University | IRB_Herman_poland.pdf |
| Portugal | the Ethical and Deontological Committee for Scientific Research (CEDIC) at the University of Lusofona | IRB_FARIAS_Portugal.pdf |
| Romania | the Institutional Review Board at the University of Pennsylvania | IRB_ETIENNE_Romania.pdf |
| Russia_1 | HSE University, Center for sociocultural research | IRB_Grigoryev_Russia.pdf |
| Russia_2 | Ethics committee of the Ural Federal University | IRB_Pavlov_Russia.pdf |
| Russia_3 | Ethics Committee of The South Ural University of Technology | IRB_Valko_Russia.pdf |
| Saudi Arabia | Research Committee at the Canadian University Dubai | IRB_Contu_UnitedArabEmirates.pdf |
| Serbia | Ethics Committee of the  Department of Psychology, Faculty of Philosophy, University  of Novi Sad | IRB_Jovanović_Serbia |
| Singapore | Human research ethics approval of the University of the Sunshine Coast | IRB_CHOW_Singapore.pdf |
| Slovakia | Univerzita Komenskeho v Bratislave | IRB_Findor_Slovakia.pdf |
| Slovenia | The Ethics Committee of the Faculty of Business, Economics and Social Sciences  of the University of Bern | IRB_BERGER_Switzerland |
| South Africa | The Ethics Committee of the Faculty of Psychology  of the University of Basel | IRB_Lagomarsino_South Africa |
| South Korea | Ulsan National Institute of Science and Technology | IRB_CHUNG_SouthKorea |
| Spain_1 | Comite Etico de Investigacion con Humanos Universidad de Cordoba | IRB_BLAYA-BURGO_Spain.pdf |
| Spain_2 | The Committee for the Use of Human Subjects in Research (CUHSR) at Esade | IRB_Schmid_Spain.pdf |
| Sri Lanka | Research Ethics at The London School of Economics and Political Sciences | IRB_Sabherwal_SriLanka.pdf |
| Sudan | Institutional Review Board at NYU Abu Dhabi | IRB_Bélanger_Morocco_Sudan.pdf |
| Sweden | IRB exempt. Waiver added to folder. | IRB_Koppel_Sweden.pdf |
| Switzerland_1 | The Ethics Committee of the Faculty of Business, Economics and Social Sciences  of the University of Bern | IRB_BERGER_Switzerland.pdf |
| Switzerland_2 | Research Ethics Commission of the University of Lausanne (CER-UNIL) | IRB_Sarrasin_Switzerland.pdf |
| Taiwan | College of Management, National Kaohsiung University of Science and Technology | IRB_NGUYEN_Taiwan.pdf |
| Tanzania | The Science-Geosciences Ethics Review Board (SG ERB) at Utrecht University | IRB_VAN DEN BROEK_KenyaTanzaniaUganda.pdf |
| Thailand | School of Global Studies, Thammasat University | IRB_GAINS_Thailand.pdf |
| Turkey_1 | Human Research Ethics Committee of Kadir Has University | IRB_OZDOGRU_Turkey.pdf |
| Turkey_2 | Human Research Ethics Committee of Kadir Has University | IRB__ULUG__TURKEY.PDF |
| UAE | Institutional Review Board at NYU Abu Dhabi | IRB_Bélanger_UAE.pdf |
| Uganda | The Science-Geosciences Ethics Review Board (SG ERB) at Utrecht University | IRB_VAN DEN BROEK_KenyaTanzaniaUganda.pdf |
| UK_1 | The University of Birmingham’s research ethics processes | IRB_BOUGUETTAYA_UNITED_KINGDOM |
| UK_2 | Science, Technology, Engineering, and Mathematics Ethical Review Committee at the University of Birmingham | IRB_CUTLER_UK.pdf |
| UK_3 | The School Research Ethics Panel | IRB_Gradidge_UK.pdf |
| UK_4 | The University of Birmingham’s research ethics processes | IRB_BOUGUETTAYA_UNITED_KINGDOM |
| Ukraine | The ethical review board at Kyiv School of Economics | IRB_BRIK_UKRAINE.pdf |
| Uruguay | Comite de Etica en Investigacion de la Facultad de Psicologia de la Universidad de la Republica | IRB_FREIRA_Uruguay.pdf |
| US_1 | Science, Technology, Engineering, and Mathematics Ethical Review Committee at the University of Birmingham | IRB_CUTLER_USA.pdf |
| US_2 | Stanford Research Compliance Office | IRB_Willer_USA |
| US_3 | The internal review board at New York University | IRB_DOELL_USA.pdf |
| Venezuela | FSW Research Ethics Review at the University of Amsterdam | IRB_ETIENNE_Venezuela.pdf |
| Vietnam | University of Economics Ho Chi Minh City | IRB_HUYNH_Vietnam |

| **Table S3:** Additional independent variables | | |
| --- | --- | --- |
| **Variable** | **Question Text** | **Type of question & possible responses** |
| Trust in climate science/scientists | 1. On average, how competent are climate change research scientists? 2. On average, how much do you trust scientific research about climate change? | Slider from 0-100 from “Not at all” to “Very much so”. Participants were also allowed to respond with “No opinion” |
| Trust in government | On average, how much do you trust your government? | Slider from 0-100 from “Not at all” to “Very much so”. Participants were also allowed to respond with “No opinion” |
| Identification as a humanitarian | To what degree do you see yourself as someone who cares about human welfare? | Slider from 0-100 from “Not at all” to “Very much so”. Participants were also allowed to respond with “No opinion” |
| Identification as a global citizen | To what degree do you think of yourself as a global citizen? | Slider from 0-100 from “Not at all” to “Very much so”. Participants were also allowed to respond with “No opinion” |
| Environmentalist Identity | To what degree…   1. do you see yourself as someone who cares about the natural environment 2. are you pleased to be someone who cares about the natural environment 3. do you feel strong ties with others who care about the natural environment 4. do you identify with others who care about the natural environment | Slider from 0-100 from “Not at all” to “Very much so”. |
| External motivation to act sustainably | Please rate the degree to which you agree/disagree with the following statements about yourself:   1. Because of today's politically correct standards, I try to appear pro-environmental. 2. I try to hide my negative thoughts about pro-environmental behavior in order to avoid negative reactions from others. 3. If I acted anti-environmental, I would be concerned that others would be angry with me. 4. I attempt to appear pro-environmental in order to avoid disapproval from others. 5. I try to act pro-environmental because of pressure from others. | Slider from 0-100 from “Strongly disagree” to “Strongly agree” |
| Internal motivation to act sustainably | Please rate the degree to which you agree/disagree with the following statements about yourself:   1. I attempt to behave pro-environmentally because it is personally important to me. 2. According to my personal values, acting non-environmental is OK. {reversed} 3. I am personally motivated by my beliefs to be pro-environmental. 4. Because of my personal values, I believe that acting anti-environmental is wrong. 5. Being pro-environmental is important to my self-concept. | Slider from 0-100 from “Strongly disagree” to “Strongly agree” |
| Pluralistic ignorance estimate | Think for a moment about people from your country and their views on climate change.  What percentage of people in your country do you think would agree with the statement "Climate change is a global emergency"? | Slider from 0-100 with the label “% Who Agree” |
| Variations of “climate change” terms^^[[1]](#footnote-1)^^ | To what degree are you willing to act to prevent {term}.  Terms included:  Climate change, global warming, global heating, the climate crisis, carbon emissions, the climate emergency, or carbon pollution | Slider from 0-100 from “Not at all” to “Very much so”. |

| **Table S4: Descriptions and sample sizes for the interventions that included additional items** | | | | | |
| --- | --- | --- | --- | --- | --- |
| **Intervention** | **Question text** | **Type of question & possible responses** | **Number of Responses** | **Number of misses (non-responses)** | **Percentage of Non-Responses (%)** |
| Work Together Norm | If you are taking steps towards reducing your carbon  footprint, to what extent would you feel like you are doing  so together with other {participant's nationality}? | Slider from 0-100 from “Not at all” to “Extremely”. Participants were also allowed to respond with “No opinion” | 5090 | 70 | 1.36 |
|  | To what extent do you feel like taking steps towards  reducing your carbon footprint is something shared with  other {participant's nationality}? | Slider from 0-100 from “Not at all” to “Extremely”. Participants were also allowed to respond with “No opinion” | 5115 | 45 | 0.87 |
|  | To what extent do you feel like taking steps towards  reducing your carbon footprint involves a sense of  togetherness with other {participant's nationality}? | Slider from 0-100 from “Not at all” to “Extremely”. Participants were also allowed to respond with “No opinion” | 5130 | 30 | 0.58 |
|  | How common do you think it is for {participant's nationality} to take steps  towards reducing their carbon footprint? | Slider from 0-100 from “Not at all” to “Extremely”. Participants were also allowed to respond with “No opinion” | 5135 | 25 | 0.48 |
|  | How often do you think {participant's nationality} make an effort towards  reducing their carbon footprint? | Slider from 0-100 from “Never” to “All the time”. Participants were also allowed to respond with “No opinion” | 5137 | 23 | 0.45 |
|  | How many {participant's nationality} do you think make an effort towards  reducing their carbon footprint? | Slider from 0-100 from “None” to “Extremely many”. Participants were also allowed to respond with “No opinion” | 5139 | 21 | 0.41 |
|  | How much do you feel like other {participant's nationality} think it is good  to take steps towards reducing their carbon footprint? | Slider from 0-100 from “Not at all” to “Extremely”. Participants were also allowed to respond with “No opinion” | 5137 | 23 | 0.45 |
|  | How much do you feel like other {participant's nationality} think you  should take steps towards reducing your carbon  footprint? | Slider from 0-100 from “Not at all” to “Extremely”. Participants were also allowed to respond with “No opinion” | 5130 | 30 | 0.58 |
|  | How strongly do you identify with your fellow {participant's nationality}? | Slider from 0-100 from “Not at all” to “Extremely”. Participants were also allowed to respond with “No opinion” | 5060 | 100 | 1.94 |
|  | How important is being {participant's nationality} to your identity? | Slider from 0-100 from “Not at all” to “Extremely”. Participants were also allowed to respond with “No opinion” | 5050 | 110 | 2.13 |
|  | How important is being similar to other {participant's nationality} to you? | Slider from 0-100 from “Not at all” to “Extremely”. Participants were also allowed to respond with “No opinion” | 5101 | 59 | 1.14 |
|  | How interested are you in reducing your carbon  emissions? | Slider from 0-100 from “Not at all” to “Extremely”. Participants were also allowed to respond with “No opinion” | 5139 | 21 | 0.41 |
|  | To what degree do you support policies aimed at  reducing greenhouse gasses? | Slider from 0-100 from “Not at all” to “Extremely”. Participants were also allowed to respond with “No opinion” | 5131 | 29 | 0.56 |
|  | To what degree would you share climate change  information on social media? | Slider from 0-100 from “Not at all” to “Extremely”. Participants were also allowed to respond with “No opinion” | 5127 | 33 | 0.64 |
|  | How interested are you in donating to tree planting  organizations? | Slider from 0-100 from “Not at all” to “Extremely”. Participants were also allowed to respond with “No opinion” | 5123 | 37 | 0.72 |
| Negative Emotions | Please rate how much of each of these emotions you  typically feel.   "When thinking about climate change, I am"   Indifferent | Slider from “Not at all” to “Very much so”. | 5067 | 100 | 1.94 |
|  | Please rate how much of each of these emotions you  typically feel.   "When thinking about climate change, I am"   Anxious | Slider from “Not at all” to “Very much so”. | 5093 | 74 | 1.43 |
|  | Please rate how much of each of these emotions you  typically feel.   "When thinking about climate change, I am"   Depressed | Slider from “Not at all” to “Very much so”. | 5055 | 112 | 2.17 |
|  | Please rate how much of each of these emotions you  typically feel.   "When thinking about climate change, I am"   Helpless | Slider from “Not at all” to “Very much so”. | 5069 | 98 | 1.90 |
|  | Please rate how much of each of these emotions you  typically feel.   "When thinking about climate change, I am"   Scared | Slider from “Not at all” to “Very much so”. | 5032 | 135 | 2.61 |
|  | Please rate how much of each of these emotions you  typically feel.   "When thinking about climate change, I am"   Guilty | Slider from “Not at all” to “Very much so”. | 5065 | 102 | 1.97 |
|  | Please rate how much of each of these emotions you  typically feel.   "When thinking about climate change, I am"   Hopeful | Slider from “Not at all” to “Very much so”. | 5050 | 117 | 2.26 |
|  | Please rate how much of each of these emotions you  typically feel.   "When thinking about climate change, I am"   Angry | Slider from “Not at all” to “Very much so”. | 5055 | 112 | 2.17 |
|  | Before reading this, did you know what the 1.5°C climate  threshold is? | Choice option between "Yes, I did know that" and "No, I did not know that". | 5167 | 0 | 0.00 |
|  | If life continues as it is (that is, with very little climate  intervention), how many years do you think we have  before we will cross the 1.5°C threshold? (please enter a  number) | Participants were expected to write a number. | 5167 | 0 | 0.00 |
|  | Please rate how much of each of these emotions you  now feel.   "When thinking about climate change, I am"   Indifferent | Slider from “Not at all” to “Very much so”. | 5081 | 86 | 1.66 |
|  | Please rate how much of each of these emotions you  now feel.   "When thinking about climate change, I am"   Anxious | Slider from “Not at all” to “Very much so”. | 5104 | 63 | 1.22 |
|  | Please rate how much of each of these emotions you  now feel.   "When thinking about climate change, I am"   Depressed | Slider from “Not at all” to “Very much so”. | 5076 | 91 | 1.76 |
|  | Please rate how much of each of these emotions you  now feel.   "When thinking about climate change, I am"   Helpless | Slider from “Not at all” to “Very much so”. | 5116 | 51 | 0.99 |
|  | Please rate how much of each of these emotions you  now feel.   "When thinking about climate change, I am"   Scared | Slider from “Not at all” to “Very much so”. | 5062 | 105 | 2.03 |
|  | Please rate how much of each of these emotions you  now feel.   "When thinking about climate change, I am"   Guilty | Slider from “Not at all” to “Very much so”. | 5094 | 73 | 1.41 |
|  | Please rate how much of each of these emotions you  now feel.   "When thinking about climate change, I am"   Hopeful | Slider from “Not at all” to “Very much so”. | 5079 | 88 | 1.70 |
|  | Please rate how much of each of these emotions you  now feel.   "When thinking about climate change, I am"   Angry | Slider from “Not at all” to “Very much so”. | 5084 | 83 | 1.61 |
| Decreasing Psychological Distance | If we take little to no climate change action, how long do  you think we have before we will reach 1.5°C / 2.7°F of  warming? (Please enter a number) | Participants were expected to write a number. | 4735 | 2 | 0.04 |
|  | Below is a list of climate change impacts. Which of these  do you think will impact where you live?   Select all that apply. | Participants were expected to choose from the following list of options (they could select multiple options)   no impact  issues for farming & crop production  inland flooding & related damages  water scarcity  increase in infectious diseases  extreme weather events  reduced animal and livestock health & productivity (e.g., heat stress, diseases, productivity & death)  reduced ocean and freshwater fish catch and lower fish-farm yields  coastal flooding & storm damage (e.g., due to cyclones, sea level rise, storm surges)  mental health issues  lack of food and nutrition  heat causing deaths, labour issues and harm from wildfires  damage to infrastructure (e.g., power, transport, communications, water and sewage systems)  damage to key economic sectors  people forced to leave their home or region (due to climate changes and extreme weather events) | 4689 | 48 | 1.01 |
|  | Please write in a few sentences: how those climate  consequences will affect you, your friends and family, and  your community.   Try to imagine these things happening today so you can  be specific and describe what it will be like. | Participants were expected to write a short text. | 4737 | 0 | 0.00 |
| Correcting Pluralistic Ignorance | Think for a moment about {participant's nationality} and their views  on climate change. How many {participant's nationality} do you  think would agree with the statement “Climate  change is a global emergency”? | Slider from 0-100 for "% Who Agree". Participants were also allowed to respond with “No opinion” | 5164 | 8 | 0.15 |
| Letter to Future Generation | Please write your letter below. You will be able to  proceed after at least 3 minutes have passed.  To remind you, this letter should describe the efforts you  are taking to ensure a more stable planet for this  family in 2050, and your personal legacy that you  want to build. | Participants were expected to write at least 100 words (5 sentences), or more, if possible. | 4040 | 4 | 0.10 |
| Future-Self Continuity | Now please write yourself a “letter from the future”. This  should be a letter you are writing in the year 2030, to your  past self. As the person that you will be in 2030, what role  would you think would be appropriate for you in respect  to climate change? What would you want to tell yourself  in the past? What would you like your past self to do? | Participants were expected to write at least 100 words (5 sentences), or more, if possible. | 4211 | 15 | 0.35 |

| **Table S5.** All variables measured in the demographics block. | | | |
| --- | --- | --- | --- |
| **Variable** | **Question Text** | **Type of question & possible responses** | **Notes** |
| Gender | “What is your gender” | Multiple choice (one answer): “Male”, “Female”, “Prefer not to say”, “Non-binary/third gender/other” | Some ethics boards required alterations of this item (see <https://osf.io/qbe84> for an overview). |
| Age | “How old are you? (please enter a number)” | Single-line text box where the content type was restricted to numerical text (in years) |  |
| Education | “How many years of formal education have you completed?” | Multiple choice (one answer): “0-6 (up to grade school)”, “7-12 (up to high school)”, “13-16 (college/undergraduate degree/certificate training)”, “More than 17 years (doctorate degree, medical degree, etc.)”, “prefer not to answer” | Some teams adapted this item differently on a country-by-country basis. This information can be found on <https://osf.io/5ypca>. |
| Political orientation | “What is your political orientation for the issues listed below? Please note, by “liberal” we mean classically left-wing, and by “conservative” we mean classically right-wing. | 2-item, 100-point, slider scale including the items “for social issues (e.g., health care, education, etc.)” and “for economic issues (e.g., taxes)” Participants were also given the option “prefer not to respond” | Due to the requirements of some internal review boards, this item was removed completely from the data acquisition in Kenya, Tanzania, and Uganda. |
| Income | “What is your total yearly family/household income?” | Multiple choice (one answer), where income was listed in 8 steps ($5,000 each), from “Less than $10,000” to “$200,000 or more”. “Prefer not to say” was also listed as a possible response. | Research teams were instructed to try to convert this scale from United States Dollars into an amount approximately representative for the country in terms of wealth distribution and type of currency. |
| Indirect SES | “Do you own/have access to these items **in your home**? (check all that apply)” | Multiple choice (multiple answers) including “Separate room for kitchen”, “Washing machine”, “Vacuum cleaner”, “Freezer/deep freeze”, “Personal computer”, “Bathroom”, “Television” |  |
| MacArthur SES ladder[^37^](https://www.zotero.org/google-docs/?OBz2h1) | **Instructions**: Think of this ladder as representing where people stand in the {country}. At the **top** of the ladder are the people who are the best off – those who have the most money, the most education, and the most respected jobs. At the **bottom** are the people who are the worst off – those who have the least money, least education, the least respected jobs, or no job. The higher up you are on this ladder, the closer you are to the people at the very top; the lower you are, the closer you are to the people at the very bottom.  **Where would you place yourself on this ladder?**  Please choose the rung where you think you stand at this time in your life relative to other people in the {country}. | Multiple choice (one answer): “Rung 10 (Top) People here are the best off”, “Rung 9”, “Rung 8”, … “Rung 1 (Bottom) People here are the worst off”. |  |
| Perceived scientific consensus | “To the best of your knowledge, what percentage of climate scientists have concluded that human-caused climate change is happening?” | Slider scale from 0 to 100, measuring the estimate (in percentage). |  |

| **Table S6: Failure rates for the second attention check by data collection team** | | | |
| --- | --- | --- | --- |
| **Team** | **Sample Size** | **Number of Second Attention Check Fails** | **Percentage of Attention Check Failure** |
| algeria | 568 | 40 | 7.04 |
| armenia | 556 | 64 | 11.51 |
| australia | 1016 | 37 | 3.64 |
| austria | 719 | 217 | 30.18 |
| belgium_1 | 762 | 126 | 16.54 |
| belgium_2 | 716 | 204 | 28.49 |
| brazil* | 1261 | 0 | 0.00 |
| bulgaria | 956 | 178 | 18.62 |
| canada_1 | 894 | 36 | 4.03 |
| canada_2 | 597 | 109 | 18.26 |
| chile | 2672 | 680 | 25.45 |
| china | 1050 | 154 | 14.67 |
| czechia | 844 | 297 | 35.19 |
| denmark | 1311 | 519 | 39.59 |
| ecuador | 1067 | 388 | 36.36 |
| finland | 734 | 109 | 14.85 |
| france | 1504 | 24 | 1.60 |
| gambia | 1073 | 546 | 50.89 |
| germany | 2355 | 810 | 34.39 |
| ghana | 837 | 315 | 37.63 |
| greece | 898 | 246 | 27.39 |
| india* | 688 | 0 | 0.00 |
| ireland | 1116 | 363 | 32.53 |
| israel | 1469 | 85 | 5.79 |
| italy_1 | 609 | 18 | 2.96 |
| italy_2 | 999 | 6 | 0.60 |
| japan_1 | 654 | 1 | 0.15 |
| japan_2 | 812 | 10 | 1.23 |
| kenya | 421 | 12 | 2.85 |
| latvia | 1545 | 1060 | 68.61 |
| mexico | 526 | 36 | 6.84 |
| morocco | 930 | 456 | 49.03 |
| netherlands_1 | 1111 | 257 | 23.13 |
| netherlands_2 | 796 | 286 | 35.93 |
| netherlands_3 | 513 | 13 | 2.53 |
| newzealand | 1037 | 32 | 3.09 |
| nigeria | 2146 | 633 | 29.50 |
| northmacedonia | 1125 | 247 | 21.96 |
| norway | 1451 | 454 | 31.29 |
| peru | 469 | 64 | 13.65 |
| philippines | 242 | 97 | 40.08 |
| poland_1 | 1911 | 28 | 1.47 |
| poland_2 | 608 | 145 | 23.85 |
| portugal | 505 | 6 | 1.19 |
| romania | 412 | 1 | 0.24 |
| russia_1 | 980 | 262 | 26.73 |
| russia_2 | 501 | 106 | 21.16 |
| russia_3 | 438 | 116 | 26.48 |
| saudiarabia | 611 | 122 | 19.97 |
| serbia | 639 | 302 | 47.26 |
| singapore | 666 | 166 | 24.92 |
| slovakia | 1257 | 230 | 18.30 |
| slovenia | 707 | 206 | 29.14 |
| southafrica | 753 | 257 | 34.13 |
| southkorea | 656 | 17 | 2.59 |
| spain_1 | 230 | 120 | 52.17 |
| spain_2 | 554 | 120 | 21.66 |
| srilanka | 513 | 100 | 19.49 |
| sudan | 1218 | 595 | 48.85 |
| sweden | 3845 | 1452 | 37.76 |
| switzerland_1 | 684 | 172 | 25.15 |
| switzerland_2 | 537 | 6 | 1.12 |
| taiwan | 256 | 50 | 19.53 |
| tanzania | 185 | 81 | 43.78 |
| thailand | 876 | 290 | 33.11 |
| turkey_1 | 623 | 264 | 42.38 |
| turkey_2 | 503 | 156 | 31.01 |
| uganda | 496 | 20 | 4.03 |
| uk_1 | 277 | 42 | 15.16 |
| uk_2 | 1469 | 517 | 35.19 |
| uk_3 | 293 | 6 | 2.05 |
| uk_4* | 501 | 0 | 0.00 |
| ukraine | 501 | 5 | 1.00 |
| unitedarabemirates | 638 | 84 | 13.17 |
| uruguay | 567 | 70 | 12.35 |
| usa_1 | 1527 | 689 | 45.12 |
| usa_2 | 2968 | 608 | 20.49 |
| usa_3 | 8518 | 3463 | 40.66 |
| venezuela | 114 | 4 | 3.51 |
| vietnam | 470 | 87 | 18.51 |
| *To note, multiple data collection teams submitted cleaned versions of their datasets, which is why some countries have 0% failure rates. | | | |

1. This item was presented in a between-subjects design. All participants in the control condition were exposed to one term exclusively. [↑](#footnote-ref-1)
